# Associations between ambient pollen exposure and measures of cognitive performance

**DOI:** 10.1097/EE9.0000000000000374

**Published:** 2025-02-25

**Authors:** Baylee Corpening, Alexandra Bürgler, Bálint Tamási, Regula Gehrig, Kexin Gan, Ana Alonso Hellweg, Axel Luyten, Sarah Glick, Minaya Beigi, Karin Hartmann, Marloes Eeftens

**Affiliations:** a Swiss Tropical and Public Health Institute, Allschwil, Switzerland; b University of Basel, Basel, Switzerland; c Federal Office of Meteorology and Climatology MeteoSwiss, Switzerland; d Division of Allergy, Department of Dermatology, University Hospital Basel and University of Basel, Basel, Switzerland; e Department of Clinical Research, University Hospital Basel and University of Basel, Basel, Switzerland; f Department of Biomedicine, University Hospital Basel and University of Basel, Basel, Switzerland

**Keywords:** Pollen, Cognitive functioning, Allergic rhinitis, Hay fever, Allergic symptoms

## Abstract

**Background:**

Prior research suggests pollen allergies may be associated with cognitive function, although the effect sizes and specific cognitive outcomes varied across studies. With pollen seasons starting earlier and intensifying due to climate change, understanding the effects of pollen exposure on cognition is increasingly relevant. This study investigated the relationship between ambient pollen exposure and cognitive performance in Swiss adults.

**Methods:**

Four cognitive assessments were used to measure the daily performance of 392 participants (299 with pollen allergies) over a 10-day period during pollen season. We used generalized additive mixed models to assess the exposure-response relationship between individualized pollen exposure and cognitive performance, as well as allergic symptom severity and cognitive performance, adjusting for individual characteristics and time-varying environmental and individual confounders (e.g., air pollution, weather, and coffee intake).

**Results:**

We did not find any statistically significant relationships between pollen exposure or severity of allergic symptoms and overall score or reaction time on tests of response inhibition, attention, and visual-spatial memory. We observed a 1% increase [95% confidence interval (CI) = 0.13%, 2.0%] in average reaction time on a test of grammatical reasoning for allergic participants at an exposure of 300 pollen/m^3^ (12-hour average).

**Conclusion:**

Overall, our study population did not exhibit changes in cognitive performance related to pollen exposure or perceived allergic symptoms. A marginal, but statistically significant effect on the response time of pollen-allergic individuals was observed for a test of verbal reasoning, however, this is likely to be a chance finding considering the number of exposures and outcomes evaluated.

**What this study adds**

Pollen seasons have been starting earlier and increasing in intensity over the last few decades due to climate change, increasing the need to develop a better understanding of the effects of pollen on human health. Previous studies have indicated that pollen exposure and pollen allergies may affect cognitive performance; however, this study has a significantly larger sample, 392 participants, and is the first to use up to 10 repeated measurements per participant to investigate the associations between pollen exposure on cognitive functioning. Our approach provides a more comprehensive understanding of the associations between ambient pollen exposure on cognition.

## Introduction

Allergic rhinoconjunctivitis triggered by pollen, hereafter referred to as pollen allergy, is an immunoglobulin E (IgE)-mediated immune response toward environmental pollen that causes inflammation of the nasal mucosa and conjunctiva.^1^ Currently, an estimated 20% of the Swiss population experiences pollen allergies, a prevalence that has increased dramatically over the last 100 years.^2,3^ Typical symptoms include nasal and eye irritation and itchiness, nasal congestion, runny nose, and sneezing,^4^ though memory troubles, difficulties with attention, and slowed processing are also often reported.^5^ Previous studies have found that individuals experiencing pollen allergies may demonstrate increased errors, slowed psychomotor processing, longer reaction times, decreased learning performance, disrupted immediate and delayed memory, and changes in attentional resources.^6–8^ In addition to the personal frustration experienced with decreased cognitive functioning, this impairment can also affect an individual’s productivity and success at school and work due to missing work days (absenteeism) and worsened performance on important tasks (presenteeism).^9,10^

The effects of pollen allergies on cognition might be mediated by changes in mood, the severity of allergic symptoms, or immune mediators involved in allergic reactions. Previous studies have demonstrated that the release of cytokines during allergic reactions may impact brain function, causing behavioral effects such as mood disruption, slowed psychomotor processing, and cognitive dysfunction.^11,12^

Overall, results from past studies assessing the associations between pollen allergies on cognition are inconsistent, as some studies have found strong indications of cognitive changes, such as slowed processing speed, increased response times, and impaired working memory,^7,8^ while others have found mild to no effects.^5^ Furthermore, the mechanisms for these cognitive changes vary, with some attributing the changes to mood and blood histamine concentration^7,8^ and others to symptom severity.^6^ Additionally, previous studies are also limited in their sample size and design, testing each participant once during and once outside of the pollen season, without considering ambient pollen concentration variations or severity of allergic symptoms.^7,8^

Over the last few decades, there have been shifts in the timing and duration of pollen seasons for several allergenic pollen types, including hazel, nettle, oak, and grasses. An increased intensity of pollen release was observed for hazel, birch, oak, beech, and nettle in Europe.^13,14^ Thus, it is increasingly important to understand the relationship between ambient pollen concentration and cognitive functioning. This project, part of a larger study called Effects of Pollen on Cardiorespiratory Health and Allergies (EPOCHAL), aims to understand the associations between ambient pollen concentration on cognitive performance. We aim to characterize the shape of the exposure-response curve between pollen concentration or severity of allergic symptoms and cognitive performance and identify whether differences in cognitive performance vary between participants who report experiencing allergic symptoms upon pollen exposure versus participants who do not.

## Methods

The design, rationale, and sample size calculations for the EPOCHAL study,^2^ as well as first results on the influence of ambient pollen concentration on symptom severity^15^ and blood pressure^16^ have been previously published. The EPOCHAL study was approved by the Ethics Committee of North-Western and Central Switzerland (EKNZ [Ethikkomission Nordwest und Zentralschweiz] number 2021-00151). Detailed inclusion and exclusion criteria and their justifications were previously published,^2,15,16^ and are briefly summarized in Table 1.

**Table 1.** Inclusion and exclusion criteria in the EPOCHAL study

Inclusion criteria	Exclusion criteria
• Adults (18–65 years) • Live in or near Basel-Stadt, Switzerland • Speak fluent German or English • Access to desktop, laptop, or tablet with internet access • Willing to undergo relevant testing and assessments (including allergy skin prick test) • Willing to refrain from recreational psychoactive drug use during assessment period	• Visual or hearing loss • Reduced cognitive capacity (such as dementia) • Pregnant individuals • Persons who have received immunotherapy for pollen within the last 5 years • Regular users of immunosuppressive medications • Individuals with serious preexisting cardiac or lung conditions, or a history of epilepsy (due to the potential risk undergoing skin prick test or spirometry)

### Recruitment of participants

Recruitment occurred in the Basel region (Switzerland) through advertisements on university-affiliated websites, social media, word of mouth, and physical flyers in the Division of Allergy of the University Hospital Basel and university notice boards. We predominantly recruited individuals with pollen allergies to better assess the associations between pollen exposure on the sensitive population. The data collection period lasted from March 2021 to September 2022.

Participants had an initial home visit with a study nurse during which the study was explained, informed consent was obtained, and an initial intake questionnaire regarding their education, employment, lifestyle, general health (including self-assessed allergic status, based on the European Community Respiratory Health Survey) and physical activity was completed.^15^ Participants who self-reported experiencing a pollen allergy will be referred to as “allergic” and the others as “nonallergic.” Participants completed a skin prick test (SPT) at the Division of Allergy of the University Hospital Basel, as detailed previously.^15,16^

### Cognitive assessments

During their typical, self-reported symptomatic pollen allergy period, participants completed online cognitive assessments once daily for 10 days, within a 14-day period. Tests were conducted via the online platform Creyos (Creyos Health, Toronto, Canada), in either English or German depending on the participant’s preference. Participants completed a practice session with a study nurse before beginning their testing period.

**Figure 1.**
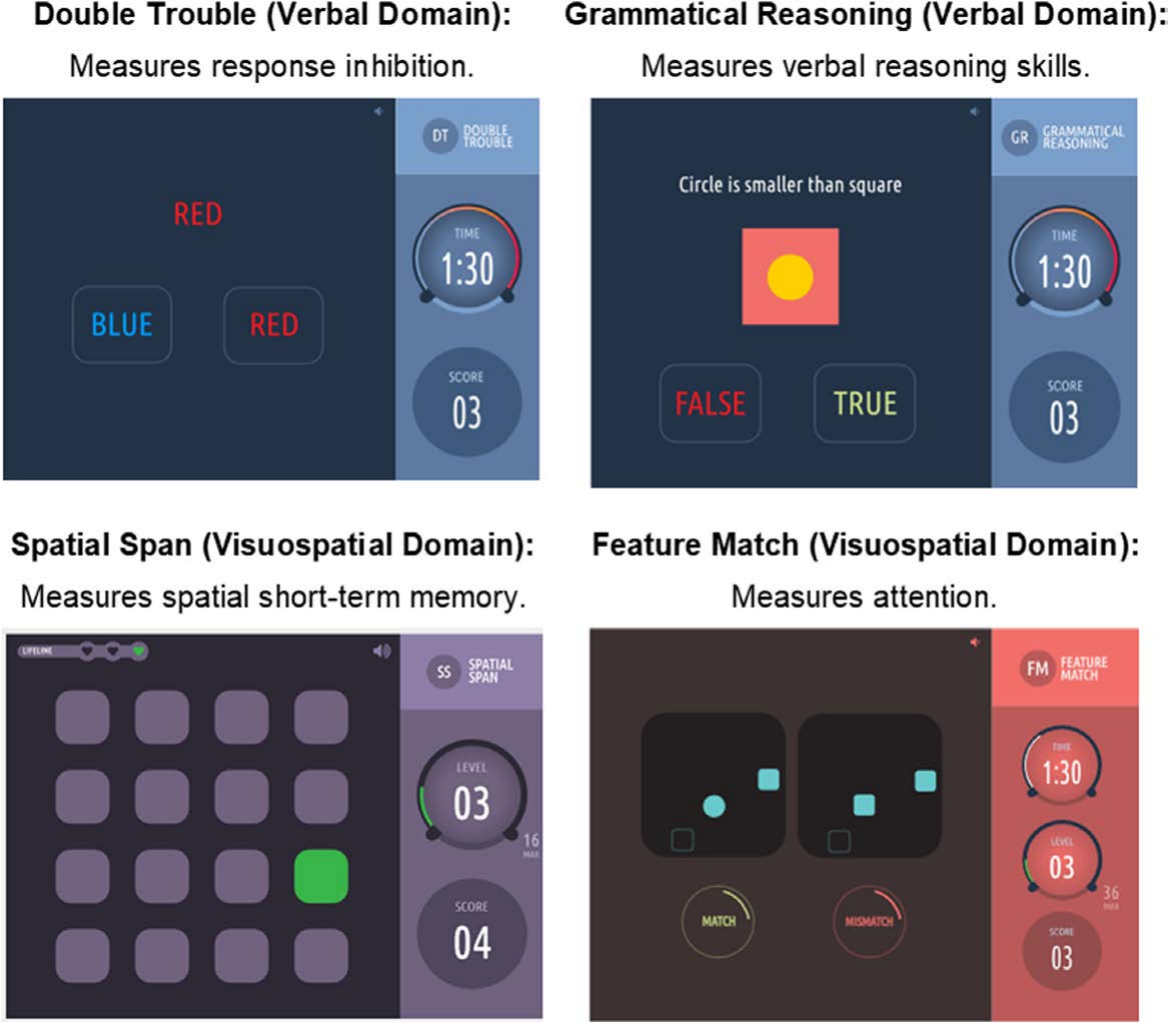
Visual summary of cognitive tests used, along with cognitive domain they assess and specific skills that are measured.

The cognitive assessments consisted of four short, game-like tests that were administered always in the same order on a participant-chosen device and took 10–15 minutes (daily) to complete. On-screen impressions of the four tests are shown in Figure 1, with more complete descriptions in Figure S.1; http://links.lww.com/EE/A331. The assessments are based on (or identical to) previously validated cognitive assessments.^17–22^ We used both overall test scores and reaction time as indicators of cognitive performance.

Invalid data (such as low scores caused by random clicking or failure to respond) were identified and excluded using Creyos validity indicators and specified disregard criteria. Details regarding disregard criteria and disregarded observations are specified in Table S.1; http://links.lww.com/EE/A331.

### Exposure assessment

**Figure 2.**
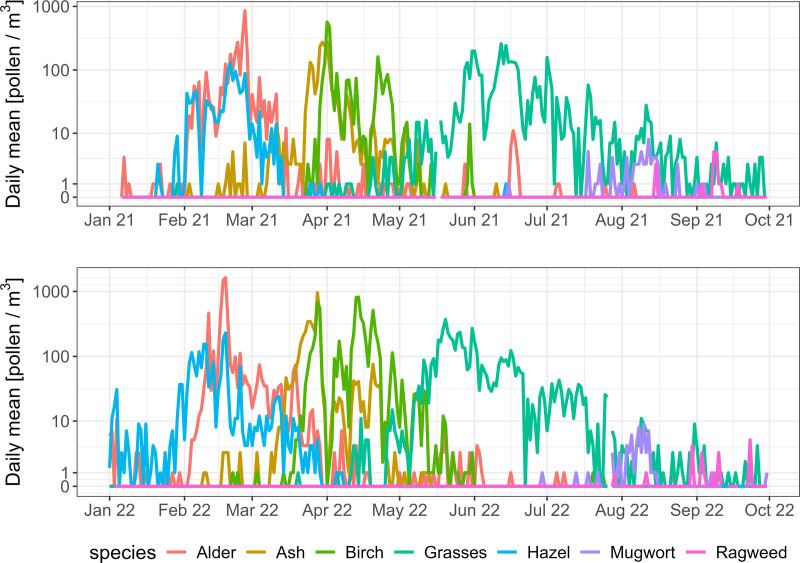
Time series of daily mean pollen concentrations in Basel (years 2021 & 2022).

Hourly ambient mean pollen concentration (pollen/m^3^), measured by a Hirst trap located in central Basel, was provided by the Federal Office of Meteorology and Climatology MeteoSwiss for the data collection period.^16^ Missing hourly data for 27 days in 2022 due to technical errors were imputed using data from the co-located automatic real-time pollen monitoring system Swisens Poleno, as outlined in Bürgler et al.^16^ Seven highly allergenic pollen types were measured: hazel, alder, birch, ash, grasses, mugwort, and ragweed. The time-varying concentration of each of these pollen types in the Basel region during the study period is shown in Figure 2.

### Potential confounders

#### Symptoms and lifestyle

Participants were asked to complete a brief daily questionnaire before starting their cognitive assessments.^23^ The daily questionnaire took measures of the participants’ health, distraction levels, alcohol and coffee consumption, medication intake, and physical activity that day. Daily medications were categorized into three groups based on the patient information leaflet, distinguishing those known to have a possible positive, negative, or no effect/unclear/unknown impact on cognitive performance. We captured allergic symptom severity with a composite nasal and ocular symptom score, in which participants rated six separate symptoms on a scale from 0 to 3 (0 = no symptoms and 3 = severe symptoms), following a validated method by the European Academy of Allergy and Clinical Immunology.^24^ Further details of these measurements were previously published.^15^

#### Environmental covariates

As environmental temperature, relative humidity, and air pollution have all been shown to have varying effects on cognition,^25–27^ and are also known to be correlated with pollen concentration, these data were collected from the Basel-Binningen monitoring station and adjusted for as potential time-varying environmental confounders.

### Statistical analysis

#### Exposure metric

In the final panel, we included individuals who completed a valid SPT and a minimum of three cognitive assessments on three separate days. Previous literature indicates that cognitive deficits related to allergy may be the result of increased cytokines released during the inflammatory response.^7,11^ To capture this potential mechanism, we used the novel metric individually-relevant pollen exposure (IPE). To calculate the IPE, the hourly mean ambient concentration of pollen types to which the participant was sensitized (confirmed via SPT) were summed for a specified time period before the cognitive assessments, as illustrated in Figure S.2; http://links.lww.com/EE/A331.^16^ Analyses of variance were used to determine within- versus between-subject variability in IPE for each outcome. For sensitivity analyses, total pollen exposure, calculated by summing the pollen concentrations for each pollen type at a given time for all participants (regardless of sensitization), was related to measures of cognitive performance.

#### Statistical models

A directed acyclic graph (Figure S.3; http://links.lww.com/EE/A331) illustrates how our model was derived. We fitted generalized additive mixed models (GAMMs) to relate cognitive assessment scores and reaction times to the average IPE over the 3, 6, 12, 24, 48, 96, and 120 hours before the start of the cognitive assessments. We fit GAMMs because they can model nonlinear exposure-response relationships and account for the dependency of within-participant measurements. We employed the GAMM function from the mgcv package (version 1.9.0) in R (version 4.3.2) to fit all models^28^ following Formula S.1; http://links.lww.com/EE/A331. For the models fitting reaction time as the outcome, we utilized the gamma family distribution with a log link function due to the data’s positive, nonzero, right-skewed nature. We modeled the score performance data assuming normally distributed errors.

To account for individual baseline differences in the repeated measurement data, a random intercept was included for each participant. We accounted for the overall population learning effect using a penalized spline for assessment day. In addition, to account for potential personal differences in learning effects, we also included personal random slopes for the assessment day. To limit model complexity and degrees of freedom used, both random intercepts and random slopes were penalized, and their coefficients were “shrunk” toward the population average. This means that, in most cases, if a participant’s personal ability or learning curve does not deviate much from the population mean, their individual effect is adjusted closer to the average.

Exposure to pollen (assessed as both IPE and total exposure) was modeled as a penalized smooth using thin plate regression splines to allow flexibility and thus nonlinearity in the modeled relationship between exposure and cognitive performance while preventing overfitting.^29^ Additionally, we conducted residual autocorrelation checks using variograms to assess and address any potential temporal correlation in the residuals.

We also used penalized smooths to adjust for the environmental variables particulate matter with less than 10 µm in diameter (PM_10_), NO_2_, relative humidity, and temperature in the 96 hours before testing, consistent with previous publications.^15,16^

Each model was adjusted for personal characteristics, such as body mass index, age, sex, level of education, and allergic status, along with time-varying personal confounders like exercise and daily intake of caffeine, alcohol, and intake of medications known to have an effect (positive or negative) on cognition (Effectively, no participants took medications that were known to affect cognitive performance positively.). We also adjusted for the hour of the day the participant completed the assessment, whether it was a weekday or weekend, and whether it was early, mid, or late pollen season.

The Akaike Information Criterion was used to determine which exposure period best fit our models for each cognitive assessment and outcome type. To explore whether associations differed depending on allergic status, we included separate penalized smooth terms for the allergic versus nonallergic group.

#### Sensitivity analyses

We performed sensitivity analyses to assess whether our personal exposure metric, IPE, was more sensitive than total pollen exposure in discerning a relationship between pollen exposure and cognitive outcomes. We also sought to validate IPE against total pollen exposure, which does not account for individual sensitizations. Additionally, models with symptom severity scores as the main predictor were used to investigate whether allergic symptoms may be the mechanism linking pollen exposure to changes in cognitive performance, as some previous literature has suggested.^6^

## Results

### Study population

We recruited 410 participants, 14 of whom did not have a valid SPT and four who did not complete the minimum of three cognitive assessments. The remaining 392 participants were included in the final analysis; 299 self-reported a pollen allergy, and 93 were nonallergic (Table 2). The cohort’s mean age at enrollment was 40 years; however, the allergic participants tended to be older than the nonallergic. While 62% of the cohort was female, the proportion of females was similar between the pollen-allergic and nonallergic groups. The study population was highly educated, 90% of the participants had a Bachelor’s degree or above. For each of the four cognitive assessments, there was an average of ten valid observations per participant.

**Table 2.** Description of the study population (overall and stratified by pollen allergy status)

Variable	Nonallergic^a^ N = 93	Allergic^a^ N = 299	Overall^a^ N = 392	*P*-value^b^
Sex				0.35
Female	62 (67%)	181 (61%)	243 (62%)	
Male	31 (33%)	118 (39%)	149 (38%)	
Age mean	37 (10)	41 (10)	40 (10)	<0.001
Age categories^c^				<0.001
18–30	25 (27%)	48 (16%)	73 (19%)	
31–40	38 (41%)	84 (28%)	122 (31%)	
41–50	17 (18%)	113 (38%)	130 (33%)	
51–65	13 (14%)	54 (18%)	67 (17%)	
BMI categories^c^				0.50
Normal weight	64 (69%)	191 (64%)	255 (65%)	
Overweight	19 (20%)	81 (27%)	100 (26%)	
Obese	10 (11%)	27 (9.0%)	37 (9.4%)	
Language				0.62
English	51 (55%)	153 (51%)	204 (52%)	
German	42 (45%)	146 (49%)	188 (48%)	
Education^d^				0.28
Below Bachelor’s degree	13 (14%)	28 (9%)	41 (10%)	
Bachelor’s degree and above	80 (86%)	271 (91%)	351 (90%)	
Employment				0.029
Full-time employment	54 (58%)	181 (61%)	235 (60%)	
Part-time employment	17 (18%)	68 (23%)	85 (22%)	
Home manager	1 (1.1%)	11 (2.8%)	10 (3.3%)	
Not currently working	5 (5.4%)	20 (6.7%)	25 (6.4%)	
Student	16 (17%)	20 (6.7%)	36 (9.2%)	
Previously physician-diagnosed pollen allergy	2 (2.2%)	192 (64%)	194 (49%)	<0.001
Pollen specific sensitization				
Alder	17 (18%)	154 (52%)	171 (44%)	<0.001
Ash	36 (39%)	181 (61%)	217 (55%)	<0.001
Birch	23 (25%)	170 (57%)	193 (49%)	<0.001
Hazel	17 (18%)	149 (50%)	166 (42%)	<0.001
Grass	19 (20%)	198 (66%)	217 (55%)	<0.001
Mugwort	23 (25%)	100 (33%)	123 (31%)	0.11
Ragweed	32 (34%)	113 (38%)	145 (37%)	0.55
Alcohol consumption^e^	11% (0.15)	12% (0.17)	12% (0.16)	0.91
Caffeine intake^e^	7% (0.12)	8% (0.18)	9% (0.16)	0.84

a Continuous variables show mean (SD), categorical variables show N (%).

b Pearson’s chi-squared test (except Age and BMI categories).

c Wilcoxon rank sum test.

d Education: Bachelor’s degree and above: Bachelor’s, Master’s or Doctoral degree at University/teacher’s college/equivalent; below Bachelor’s degree: Primary school, Lower secondary education, Higher secondary education, grammar school; Postsecondary, nontertiary education.

e Percentage of assessments completed within 4 hours of participant reported consumption of alcohol, or 2 hours of consumption of caffeine.

BMI, body mass index.

Table 3 provides an overview on the mean personal average scores and mean personal average reaction times for each test. There was no significant difference in average scores between the allergic and nonallergic groups for any of the tests. We did find a statistically significant difference in reaction time between the allergic group and the nonallergic group on the Double Trouble test and Feature Match test. The allergic group performed an average of 106 milliseconds slower on the Double Trouble test, and 99 milliseconds slower on the Feature Match test than the non-allergic group.

**Table 3.** Average test scores and reaction times stratified by pollen allergy status

Outcome measure	Nonallergic N = 93	Allergic N = 299	Overall N = 392	*P* value
Average score				
Double trouble	48 (12)	45 (13)	46 (13)	0.1
Feature match	139 (21)	139 (22)	139 (21)	0.97
Grammatical reasoning	18.9 (4.6)	18.5 (4.9)	18.6 (4.8)	0.69
Spatial span	5.99 (0.73)	5.88 (0.79)	5.90 (0.78)	0.26
Average reaction time (milliseconds):				
Double trouble	1800 (428)	1906 (482)	1880 (472)	0.049
Feature match	2931 (399)	3030 (391)	3007 (395)	0.043
Grammatical reasoning	3482 (791)	3588 (904)	3563 (879)	0.46
Spatial span	11382 (1540)	11359 (1321)	11365 (1374)	0.91

Scores and reaction times are presented as mean personal averages (SD), specifically the means of the individual participants’ means. P-values are derived from Pearson’s chi-squared test.

### Main findings

#### Exposure period

We compared models with different exposure periods to determine which averaged exposure period affected cognitive performance most. The evaluated exposure periods (i.e. periods before the cognitive tests) were 3, 6, 12, 24, 48, 72, 96, and 120 hours. Using the Akaike Information Criterion to assess model fit, we found a lack of consistency for the exposure averaging periods that obtained the best fits, varying between 3 and 120 hours (Table 4). Within- versus between-subject variability for IPE (in % of the total) is also displayed in Table 4 for each outcome.

**Table 4.** Best fitting exposure periods (averaged pollen exposure in the duration before beginning cognitive tests) for each assessment-outcome pair, as determined using lowest Akaike Information Criterion, as well as within/between subject variability

Cognitive assessment:	Overall score outcome		Reaction time outcome	
	Best fitting exposure period	Within/between subject variability IPE	Best fitting exposure period	Within/ between subject variability IPE
Double trouble	3 hours	70.2%/29.8%	12 hours	69.6%/30.4%
Feature match	120 hours	43.7%/56.3%	96 hours	48.5%/51.5%
Grammatical reasoning	3 hours	70.3%/29.7%	12 hours	69.8%/30.2%
Spatial span	3 hours	70.2%/29.8%	120 hours	43.6%/56.4%

#### Cognitive performance

Using the best-fitting exposure periods, we found no significant relationship between pollen exposure and cognitive test scores for any of the four cognitive assessments. Table 5 shows the estimated difference in the overall score for each cognitive assessment at an IPE and total pollen exposure of 300 pollen/m^3^ (defined as a high exposure for most of our pollen species) compared to no pollen exposure. Additionally, Table 5 includes the estimated difference in absolute and relative test scores when an interaction with allergy status is included, delineating between allergic and nonallergic individuals. Concentration-response curves are included in supplemental Figures S.4A–D; http://links.lww.com/EE/A331. Adjustment for learning effects was highly important for all tests, with test scores improving between 5.3% (spatial span task) and 55.1% (double trouble task) and average reaction time decreasing significantly for all tasks except spatial span (supplemental Figures S.5A and S.5B; http://links.lww.com/EE/A331, respectively). Learning effects were much larger than both test score and reaction time differences associated with changes in either IPE or total pollen exposure.

**Table 5.** Difference in absolute and relative overall test scores of each cognitive assessment, for the overall study population and stratified by allergic status, at 300 pollen/m^3^ IPE, and 300 pollen/m^3^ total pollen exposure

Cognitive test	Difference in score at 300 pollen/m^3^ IPE		Difference in score at 300 pollen/m^3^ total pollen exposure	
	Absolute	Percentage	Absolute	Percentage
Double trouble	0.41 (−0.43, 1.26)	0.89% (−0.93%, 2.74%)	−0.10 (−0.27, 0.07)	−0.22% (−0.59%, 0.15%)
Allergic	0.38 (−0.49, 1.25)	0.84% (−1.09%, 2.78%)	−0.18 (−0.39, 0.02)	−0.40% (−0.87%, 0.04%)
Nonallergic	0.48 (−2.31, 3.26)	1.00% (−4.81%, 6.79%)	0.06 (−0.22, 0.34)	0.13% (−0.46%, 0.71%)
Feature match	−0.45 (−3.51, 2.60)	−0.32% (−2.53%, 1.87%)	0.68 (−1.62, 2.98)	0.49% (−1.17%, 2.14%)
Allergic	2.34 (−3.52, 8.20)	1.68% (−2.53%, 5.90%)	−0.87 (−6.37, 4.64)	−0.63% (−4.58%, 3.34%)
Nonallergic	−2.69 (−19.9, 14.5)	−1.94% (−14.3%, 10.4%)	10.1 (1.46, 18.7)*	7.27% (1.05%, 13.5%)*
Grammatical reasoning	0.11 (−0.03, 0.25)	0.59% (−0.16%, 1.34%)	0.04 (−0.02, 0.10)	0.22% (0.11%, 0.54%)
Allergic	0.16 (−0.02, 0.34)	0.86% (−0.11%, 1.84%)	0.04 (−0.08, 0.16)	0.22% (−0.43%, 0.86%)
Nonallergic	−1.29 (−2.99, 0.42)	−6.83% (−15.82%, 2.22%)	−0.01 (−0.37, 0.36)	−0.05% (−1.96%, 1.90%)
Spatial span	0.01 (−0.06, 0.08)	0.17% (−1.02%, 1.36%)	−0.01 (−0.03, 0.01)	−0.17% (−0.51%, 0.17%)
Allergic	0.02 (−0.06, 0.10)	0.34% (−1.02%, 1.70%)	−0.01 (−0.04, 0.02)	−0.17% (−0.68%, 0.34%)
Non−allergic	−0.21 (−0.57, 0.15)	−3.51% (−9.52%, 2.50%)	−0.004 (−0.04, 0.03)	0.0% (−0.67%, 0.50%)

**Figure 3.**
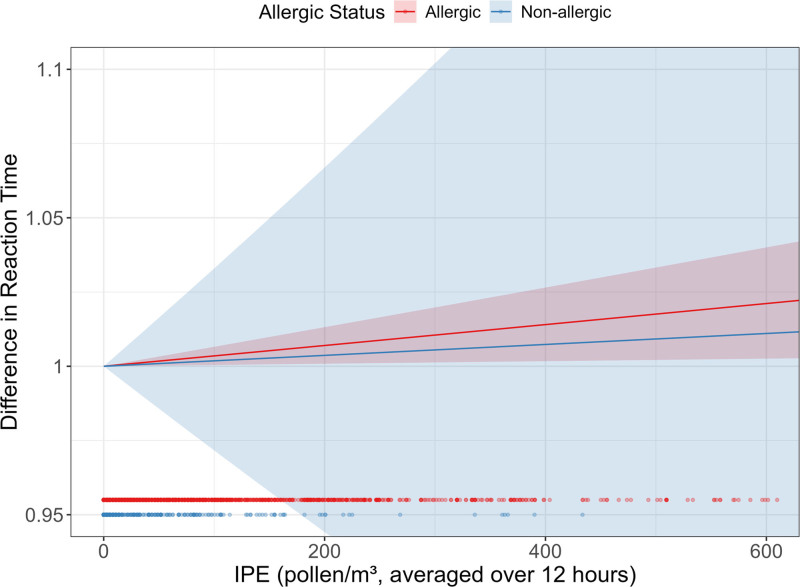
Change in grammatical reasoning reaction time relative to individually relevant pollen exposure (IPE): The plot depicts the change in Grammatical Reasoning reaction time (on a multiplicative scale) in relation to IPE, averaged over a 12-hour period, stratified by allergic status. The bottom dotted lines show the distribution of observations for each group. Shaded regions indicate the 95% confidence intervals for each group.

Table 6 shows the estimated difference in average reaction time for each cognitive assessment at an IPE and total pollen exposure of 300 pollen/m^3^ compared to 0 pollen/m^3^ for the full population and for the allergic and nonallergic subgroups. For the cognitive assessment the Grammatical Reasoning, there was a 1% increase in average reaction time, which was statistically significant. Further, when we adjusted for allergic status, allergic individuals had a 1% increase in reaction time, while nonallergic individuals did not. Figure 3 visualizes this relationship. Reaction time for the other tests did not differ significantly between a high and a no-exposure scenario (Figures S.6A−C; http://links.lww.com/EE/A331).

**Table 6.** Percentage difference in reaction times for each cognitive assessment, for overall study population and stratified by allergic status, at 300 pollen/m^3^ IPE, and 300 pollen/m^3^ total pollen exposure

Cognitive test	Estimated percent difference in reaction time at 300 pollen/m^3^ IPE	Estimated percent difference in reaction time at 300 pollen/m^3^ total pollen exposure
Double trouble	0.3% (−2.0%, 2.0%)	0.5% (−0.9%, 2.0%)
Allergic	0.12% (−2.0%, 2.3%)	0.1% (−1.5%, 1.7%)
Nonallergic	3.1% (−6.0%, 13.0%)	0.5% (−1.0%, 1.8%)
Feature match	0.5% (−1.3%, 2.3%)	0.4% (−1.0%, 1.7%)
Allergic	0.4% (−1.4%, 2.2%)	0.01% (−0.03%, 0.04%)
Nonallergic	4.3% (−6.5%, 16.4%)	0.1% (−0.1%, 0.3%)
Grammatical reasoning	1.0% (0.1%, 2.0%)*	0.6% (0.01%, 1.3%)*
Allergic	1.0% (0.13%, 2.0%)*	0.87% (0.16% 1.6%)*
Nonallergic	5.5% (−8.3%, 10.2%)	−1.9% (−4.4%, 0.65%)
Spatial Span	−1.0% (−2.4%, 0.4%)	−1.0% (−2.0%, 0.1%)
Allergic	−1.0% (−2.4%, 0.5%)	−1.0% (−2.0%, 0.1%)
Nonallergic	−4.3% (−11.7%, 3.8%)	−1.0% (−3.0%, 1.6%)

*Indicates a statistically significant association.

#### Sensitivity analysis

##### Exposure metric

We consistently found null results regardless of whether IPE or total pollen exposure was used as the exposure metric. Using total pollen exposure, there were still no significant relationships observed with test scores for any of the cognitive assessments, nor with reaction time in the double trouble, feature match, and spatial span tasks. There was still a statistically significant increase in reaction time for the grammatical reasoning assessment, though the effect size at 300 pollen/m^3^ was slightly smaller (an increased reaction time of 0.6% rather than 1%) than the effect size when using IPE.

##### Symptom severity score

**Figure 4.**
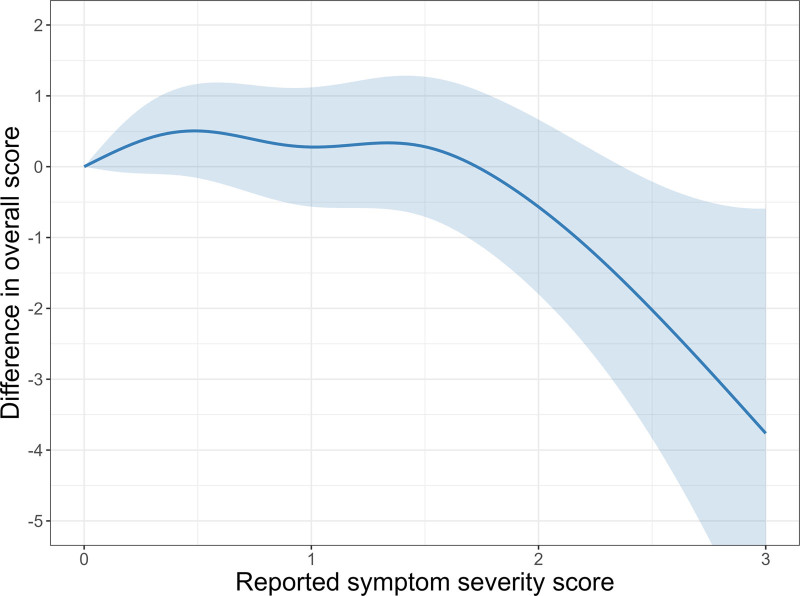
Change in double trouble overall score relative to reported symptom severity score for the full study population (n = 392). The shaded area represents the 95% confidence interval.

Self-reported nasal-ocular symptom severity did not appear to be significantly associated with score performance or reaction time for feature match, grammatical reasoning, or spatial span. For the double trouble assessment, we did not observe a decrease in performance between no, mild, and moderate symptom severity (symptom scores 0–2), however, there was a significant decrease in overall score observed at the highest symptom severity (Figure 4). However, data points in the severe symptom score range were rather sparse. We provide further concentration-response plots for associations with overall scores in supplemental Figures S.7A–C; http://links.lww.com/EE/A331 and for associations with reaction time in supplemental Figures S.8A–D; http://links.lww.com/EE/A331.

## Discussion

### Main associations

Our results indicated no significant relationship between individually-relevant pollen exposure and overall scores on four cognitive assessments, regardless of allergic status. However, allergic participants showed slightly slower average reaction times on a grammatical reasoning test at high pollen concentrations. This effect was smaller but still present when using total pollen exposure.

Minimal performance changes and stable reaction times are consistent with past literature, though the results from previous studies tend to vary in the size and consistency of their observed effect and concern different aspects of cognitive performance. For instance, Trikojat et al.^8^ observed that allergic patients demonstrated a significantly slower reaction time (120 milliseconds) on a test of dual task processing during the allergy season when compared with the nonallergy season, while Marshall et al.^5^ found a small increase in processing reaction time for allergic participants only under one testing condition, and for only one of the tests in their battery. Wilken et al.^30^ reported that symptomatic individuals had slower response times when solving math calculations after exposure to ragweed pollen in a controlled setting; however, their performance accuracy did not change. Finally, Papapostolou et al.^6^ found a negative correlation between allergic symptom scores and reaction time for simple movement in allergic children, indicating that symptom severity may have a mediating effect on reaction time.

While processing speed and reaction times may be affected by allergic rhinitis and elevated pollen concentration for some cognitive domains, our study did not observe a clear difference in reaction time across all cognitive tests. Moreover, the difference we observed (a 1% increase, or approximately 36 milliseconds slower for allergic participants at 300 pollen/m^3^) was relatively small. This may reflect that the effect is not as strong as some previous literature indicates, or that slowed processing may occur in different cognitive domains than the verbal and visuospatial domains tested here.

In contrast to our results, previous studies reported worse performance on some memory tasks for allergic individuals. For example, Trikojat et al.^8^ noted difficulties with short-term verbal memory while experiencing allergic inflammation, and Marshall et al.^5^ reported that allergic participants performed worse during the allergy season on a test of verbal working memory (though they found no difference in performance on several other tasks in a battery of working memory tests). Papapostolou et al.^6^ found an increase in errors on a visual-spatial working memory task in allergic children during the allergy season compared with matched controls. Because our study used a different spatial memory test, the results are not directly comparable with each of the findings above.

Whereas Papapostolou et al.^6^ found that symptom severity was associated with worse performance, we did not find decreased performance or changes in reaction times related to symptom severity, except in the double trouble task, where there was a significant decrease in overall score performance associated with severe self-reported symptoms. This decrease was not observed at mild or moderate self-reported symptoms. However, due to the limited number of participants who reported severe nasal and ocular symptoms, we should be cautious about drawing firm conclusions regarding this association.

Several factors may explain our null results regarding overall cognitive performance. Online neuropsychological assessments, while increasingly used in research settings due to their efficiency, are not always a perfect substitute for traditional neuropsychological exams.^31^ For example, they have been shown to be less reliable in capturing reaction times due to differences in social pressure and engagement, distracting testing environments, and differences in digital resources.^31^ We attempted to mitigate these effects by asking participants to use consistent testing setups and avoid distractions.

Furthermore, many neuropsychological examinations were created with the intent of diagnosing neurological conditions and may be less sensitive to minor cognitive fluctuations observed in otherwise healthy adults caused by pollen exposure and allergic inflammation.^32^ The high education level of our study population may also result in less fluctuation compared with a more representative sample, as education has been positively correlated with cognitive functioning.^33^ This higher “cognitive reserve”^34^ has been shown to act as a protective factor for cognitive functioning in cases of mild cognitive impairment,^35^ Alzheimer’s,^36^ multiple sclerosis^37^ and stroke events in young patients.^38^

A perception of cognitive performance diminution among adults with pollen allergy may also explain why this symptom is self-reported but not corroborated with objectively measured differences on cognitive tests. One study, which performed randomized, double-blind trials to assess the perception of cognitive performance versus objective performance in a population of healthy individuals, individuals with epilepsy, and individuals with Parkinson’s disease, found that for each population, the subjects’ perception of their cognitive performance was highly correlated to their mood rather than their objective performance.^39^ This may indicate that allergic individuals who report diminished cognitive capabilities while experiencing allergies may not be performing objectively worse but are rather experiencing perceptual differences in cognition related to mood. In support of this possibility, high pollen concentrations have been associated with worsening mood, and seasonal allergies have been associated with a higher risk of mood disorders.^40–42^ The relationship between pollen exposure, mood, and cognition is speculative, and further research is needed to understand how, and if, these factors may be affecting each other.

### Relevant exposure period

We did not identify a single consistent exposure averaging period as universally relevant for the four cognitive assessments, as the best-fitting exposure periods varied between 3 and 120 hours. This inconsistency likely reflects the absence of a strong relationship between pollen exposure and cognitive performance, reinforcing our null findings and suggesting there is no clear link between pollen concentration and the cognitive outcome metrics.

### Strengths and limitations

A key strength of this study is the use of hourly pollen measurements and repeated cognitive measurements over a 10-day period. This allowed for a more informative understanding of the participants’ daily cognitive fluctuations, as well as a possible exposure-response relationship between pollen concentration and cognitive performance. Previous studies^7,8^ performed cognitive testing only once during and once outside of pollen season, which may have limited their ability to account for other time-varying confounders, including other air quality variables (PM_10_ and NO_2_), fluctuating pollen concentration within the pollen season, and daily behavioral variations (caffeine, alcohol, exercise, and medication intake) which may affect cognitive performance.

In line with previous studies,^7,8,30^ this study relied on SPT to assess allergy sensitization profiles for each participant. Uniquely to this study, we used these profiles to determine the IPE, thereby focusing on the personally relevant pollen exposure for each participant during their participation in the cognitive testing. This allowed for a more individualized exposure assessment, rather than considering total pollen exposure regardless of sensitization. This decision was made under the assumption that the inflammatory response is a part of the mechanism influencing cognition.^11^ Modeling an interaction with allergy status, as well as modeling both IPE and total pollen exposure, allowed us to asses and validate this novel exposure metric. Generally, we found null results regardless of the exposure metric, but the effect size for the difference in reaction times on the grammatical reasoning test was slightly larger using IPE rather than total pollen exposure.

Further, our IPE metric weights all seven pollen types equally as long as the individual demonstrates a sensitization on SPT, though it is possible that their symptomatic response following real-world exposure varies. Further, allergenicity can be affected by meteorological and environmental factors that vary in space and time, even when the pollen concentration is stable.^43^ Despite this, IPE has been shown to be predictive of symptoms and useful in assessing the associations between pollen with other biological processes (such as cardiovascular health), demonstrating its value as a proxy for assessing the associations with cognitive functioning.^15,16^

We used a single, centrally located pollen measurement station in Basel to collect pollen concentration data, which may not perfectly reflect the pollen exposure for each participant due to variations within the city, microclimates, and individual behavior. Study participants lived a median distance of 2.3 km (interquartile range: 1.1–4.8 km) from the pollen trap, located centrally on the University hospital’s rooftop. Due to the sparse distribution of routine pollen monitors, few studies have attempted to assess local, within-city pollen contrasts.^44,45^ While some spatial differences have been observed, pollen traps are generally considered to be regionally representative.^44^ Within Switzerland, we find typically high temporal correlations between daily pollen concentrations measured in Basel and in other nearby cities.^46^ While we considered possible distractions, physical activity, alcohol and coffee consumption, and medication intake, we could only assess a limited number of time-varying confounders daily. We did not assess the use of air purifiers, window opening, or other exposure-modifying behaviors. Although we assessed time spent outdoors, we did not ultimately include it in the model because of potentially opposing effects on individuals with and without pollen allergies. While time outdoors is generally beneficial for cognition,^47^ this may not be the case for allergic individuals, who may stay indoors on high-pollen days.

Finally, the study’s volunteer-based population was highly educated, with 90% holding a Bachelor’s degree or higher, which may affect cognitive performance trends and limit generalizability. However, repeated measurements allowed participants to act as their own controls, accounting for individual differences and providing a reliable assessment of exposure associations.

## Conclusion

We did not find evidence that increased pollen exposure is associated with decreased performance on cognitive assessments. We did find an increase in reaction time for a verbal reasoning assessment for participants with pollen allergy at a high level of pollen exposure. As this effect was small, and limited to a single cognitive test, we cannot conclude that increased pollen exposure broadly affects reaction time across multiple cognitive domains. We observed worse performance on a test of response inhibition for individuals experiencing severe allergy symptoms, however, we did not observe a change in performance for individuals experiencing mild to moderate symptoms. Our findings suggest that there may not be a strong relationship between pollen exposure and cognitive performance when using online cognitive tests as objective performance indicators. This finding is corroborated by the inconsistency of results in the current literature. However, as cognitive issues are commonly reported as a symptom of pollen allergies, further exploration into the subjective perception of cognition, as well as studies using more comprehensive and sensitive cognitive test batteries, is warranted.

## Conflicts of interest statement

The authors declare that they have no conflicts of interest with regard to the content of this report.

## ACKNOWLEDGMENTS

We thank our study nurses and study assistants Alexandra Plattner, Marianne Rutschi, Claire Dexter, Judith Boegli, Jutta Gerber, Katharina Mäder, Cornelia Mathis, Margarethe Wiedenmann, Luc Schuler, and Mauritz Bürgler for their efforts in recruitment, data collection, and logistical planning. We acknowledge Astrid Baumgartner for her help with planning skin prick tests, as well as Moira Rathgeb for her skillful assistance with skin prick tests at the Division of Allergy, Department of Dermatology, University Hospital Basel.

## Supplementary Material


