# Supplementary material for: Associations between ambient pollen exposure and measures of cognitive performance
Source: Environ Epidemiol. 2025 Feb 25;9(2):e374. doi: 10.1097/EE9.0000000000000374 (PMC11864305; doi:10.1097/EE9.0000000000000374)
Supplement: Supplementary file 1 [file ee9-9-e374-s001.pdf]

Supplementary materials: Associations between ambient pollen exposure and measures of cognitive performance

Baylee Corpening[1,2], Alexandra Bürgler[1,2], Bálint Tamási[1,2], Regula Gehrig[3], Kexin Gan[1,2], Ana Alonso Hellweg[1,2], Axel Luyten[1,2], Sarah Glick[1,2], Minaya Beigi[4], Karin Hartmann[4,5,6], Marloes Eeftens[1,2]

Affiliations:

1. Swiss Tropical and Public Health Institute, Allschwil, Switzerland
2. University of Basel, Basel, Switzerland
3. Federal Office of Meteorology and Climatology MeteoSwiss, Switzerland
4. Division of Allergy, Department of Dermatology, University Hospital Basel and University of Basel, Basel, Switzerland
5. Department of Clinical Research, University Hospital Basel and University of Basel, Basel, Switzerland
6. Department of Biomedicine, University Hospital Basel and University of Basel, Basel, Switzerland

Corresponding author:
Marloes Eeftens
marloes.eeftens@unibas.ch

Department of Epidemiology and Public Health
Swiss Tropical and Public Health Institute
Kreuzstrasse 2, 4123 Allschwil, Switzerland

Telephone: +41 61 284 87 25
Fax: +41 61 284 81 05

Double Trouble (Verbal Domain):

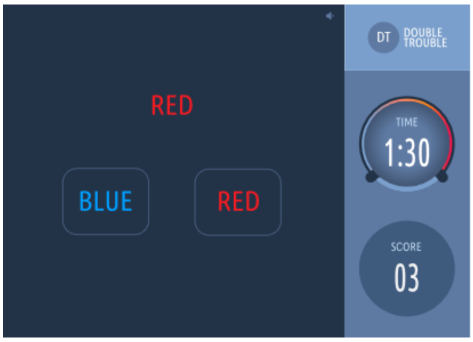

This test measures response inhibition and sustained attention, and is based on the Stroop color-word task [1]. On this test, participants were presented either the word “BLUE” or “RED,” randomly colored in either red or blue, along with two possible responses (“BLUE” and “RED”) also randomly colored in either a shade of blue or red. Participants were tasked with choosing the word that correctly described the color of the stimulus word, while disregarding the actual word and the color of the response options. The challenge was to focus on the relevant information while ignoring the irrelevant distractions. Participants had 90 seconds to provide as many correct answers as possible, and incorrect responses were deducted from their final score.

Feature Match (Visuospatial Domain):

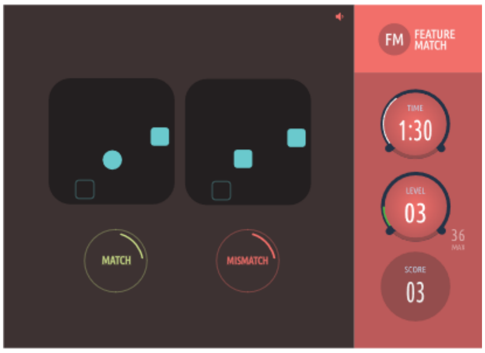

This attention-measuring test was inspired by an attention task used in fMRI studies, in which participants had to distinguish whether two stimuli were identical or not [2]. Particpants were shown two figures that were either identical or slightly differing and asked to determine if they were matching or mismatching. They had 90 seconds to answer as many questions as possible. The task increased in difficulty with each question, the more difficult questions worth more points. However, incorrect responses deducted points from the final score. This task required sustained attention to a visual processing task that increased in difficulty over time.

Grammatical Reasoning (Verbal Domain):

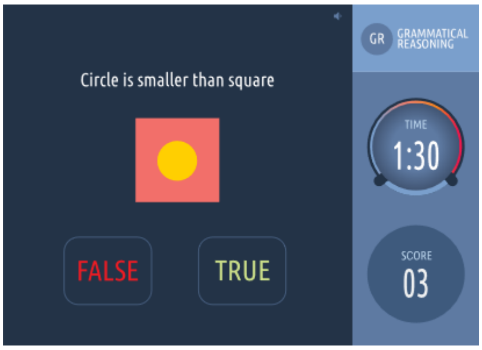

This test assessed verbal reasoning skills and was adapted from Alan Baddeley’s ‘Grammatical Reasoning Test’ [3]. Participants were presented with a figure and a sentence that either correctly or incorrectly described the figure. They were then required to determine if the statement was true or false. Participants had 90 seconds to provide as many responses as possible, incorrect responses were deducted from the final score. The test required participants to read complex statements, mentally evaluate the content, and quickly make assessments about their accuracy.

Spatial Span (Visuospatial Domain):

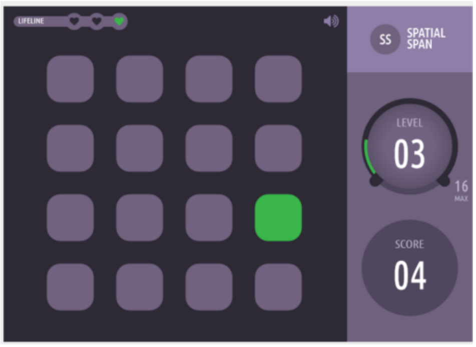

Measuring spatial short-term working memory, this test was based on the Corsi Block Tapping task [4]. Participants were shown a grid of boxes that flashed green one at a time in a random sequence. Their task was to observe and remember the sequence, including the location and order of the boxes, and then replicate it. The task began with four flashing boxes, and the difficulty increased or decreased as the participants succeeded or failed, until the participant made three incorrect guesses. There was no time limit and the final score was equal to highest number of boxes the participants were able to correcly recall.

Figure S.1 *Description of cognitive Assessments*

Table S.1 Disregardal Criteria. *Creyos uses validity indicators, which vary by task, to mark unusual performance and indicate potentially invalid results. Data marked as invalid were disregarded.*

Test Name	***Observations deemed invalid from Creyos criteria***	***Observations deemed invalid using study team criteria***
Double Trouble	29	71
Feature Match	11	1
Grammatical Reasoning	36	7
Spatial Span	36	0

To be sure of data validity, the study team decided that observations with scores over two standard deviations below average were also disregarded if they also reported at least one of the following:

1. *It was their first time completing the tests*
2. *They reported a cognitive distraction on their daily questionnaire*
3. *They consumed alcohol prior to testing, as reported in their daily questionnaire*
4. *Their personal average was over two standard deviations below panel average (indicating that they may not have understood the test during their testing period)*

Because some participants struggled to understand the rules of the Double Trouble test, 386 out of 392 participants still had at least three valid Double Trouble test observations after disregarding invalid data, while all participants still had sufficient valid data for the other three tests. Out of the four cognitive assessments used, there was an average of 10.02 valid observations per participant for Double Trouble, 10.09 valid observations per participant for Feature Match, 10.04 valid observations per participant for Grammatical Reasoning, and 10.04 valid observations per participant for Spatial Span.

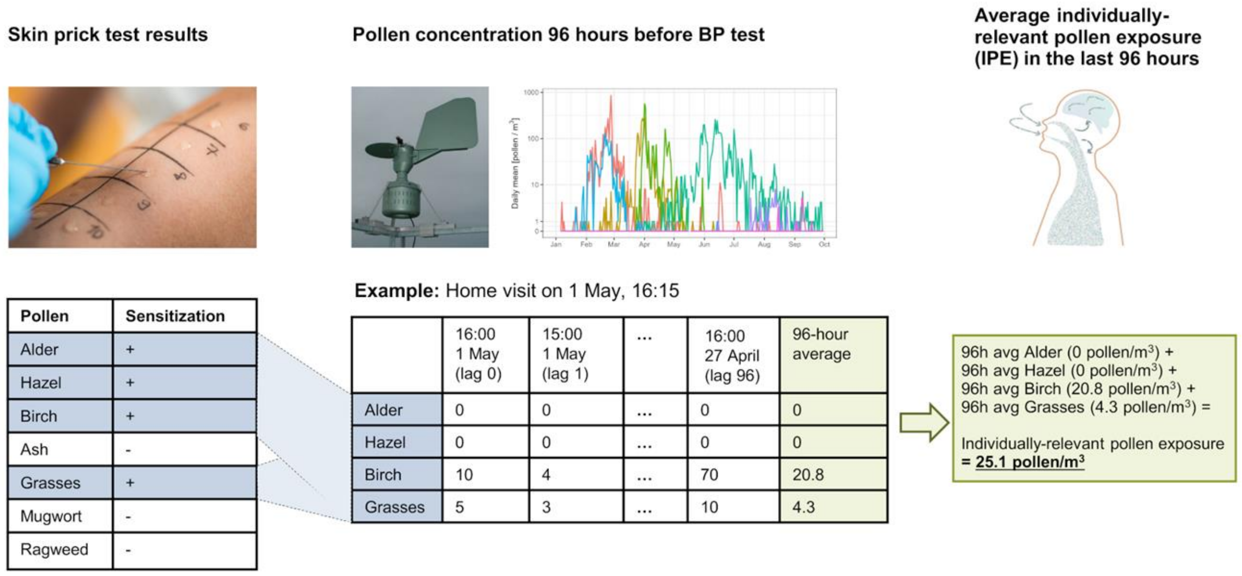

Figure S.2 Individual Pollen Exposure Metric. *This figure is taken from a previous EPOCHAL publication [5] and demonstrates how the individually-relevant pollen exposure metric is calculated*

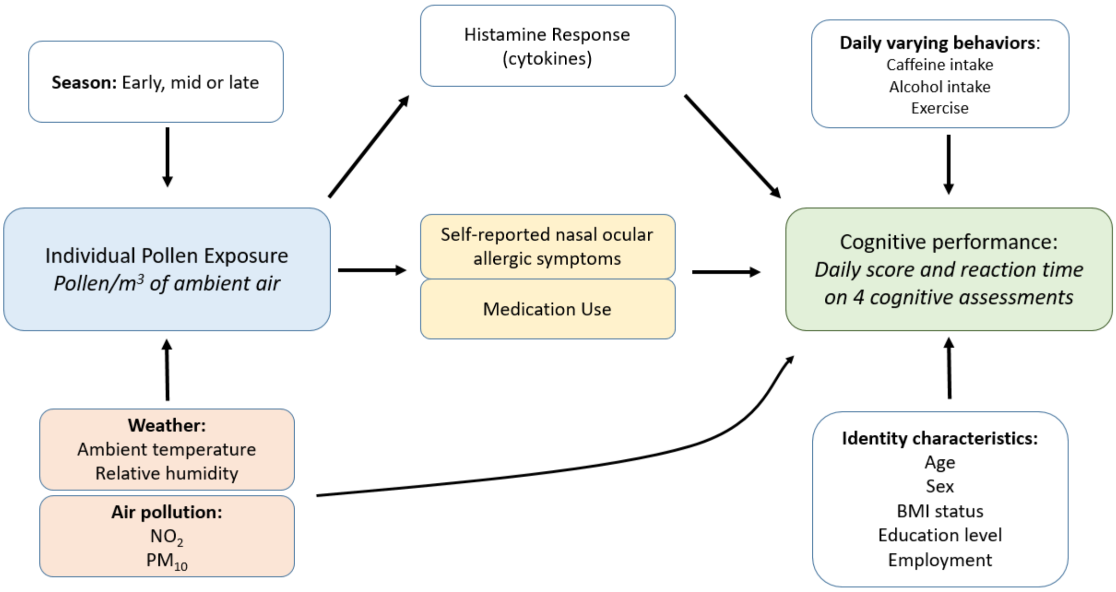

Figure S.3 Directed Acyclic Graph. *This Directed Acyclic Graph includes all modeled variables. The exposure of interest is shaded in blue, and the outcomes of interest are shaded in green. Shaded in yellow are variables that may mediate the pathway through which pollen exposure affects cognitive performance. Nasal ocular symptoms are self-reported and reflect whether the participant experienced symptoms due to allergies. Shaded in orange are environmental confounders that may affect both pollen concentration and cognitive performance. The non-shaded variables depict behaviors, characteristics or other factors that were variable to each participant which may have had an impact on their exposure or their outcome, and which were corrected for in the models. The histamine response was accounted for using the IPE metric as a proxy indication.*

Formula S.1 Modelling Overview.

Model Formula:

gamm(Cognitive outcome[a] ~ s(Pollen[b], by = strata[c]) +

s(PM_{10}) + s(NO_2) + s(relative humidity) + s(ambient temperature) +

caffeine intake + alcohol intake + cognition lowering medications + exercise +

weekday/weekend + s(hour of the day, bs=”cc”[d]) + s(assessment number) +

allergic status + sex + age + BMI status + education + season +

random = list(pid = ~ 1 + assessment day)[e],

knots = list(hour = c(0,24)[d])

)

where:

a takes the overall score or reaction time of each of four cognitive tests

b can be either IPE or total pollen

c is the strata allergic status

d is the definition of a cyclic cubic spline for hour of the day, where 0 and 24 are the same hour

e is the defintion of an individual random intercept and slope for assessment day

S.4 Difference in overall scores for each test using individually-relevant pollen exposure

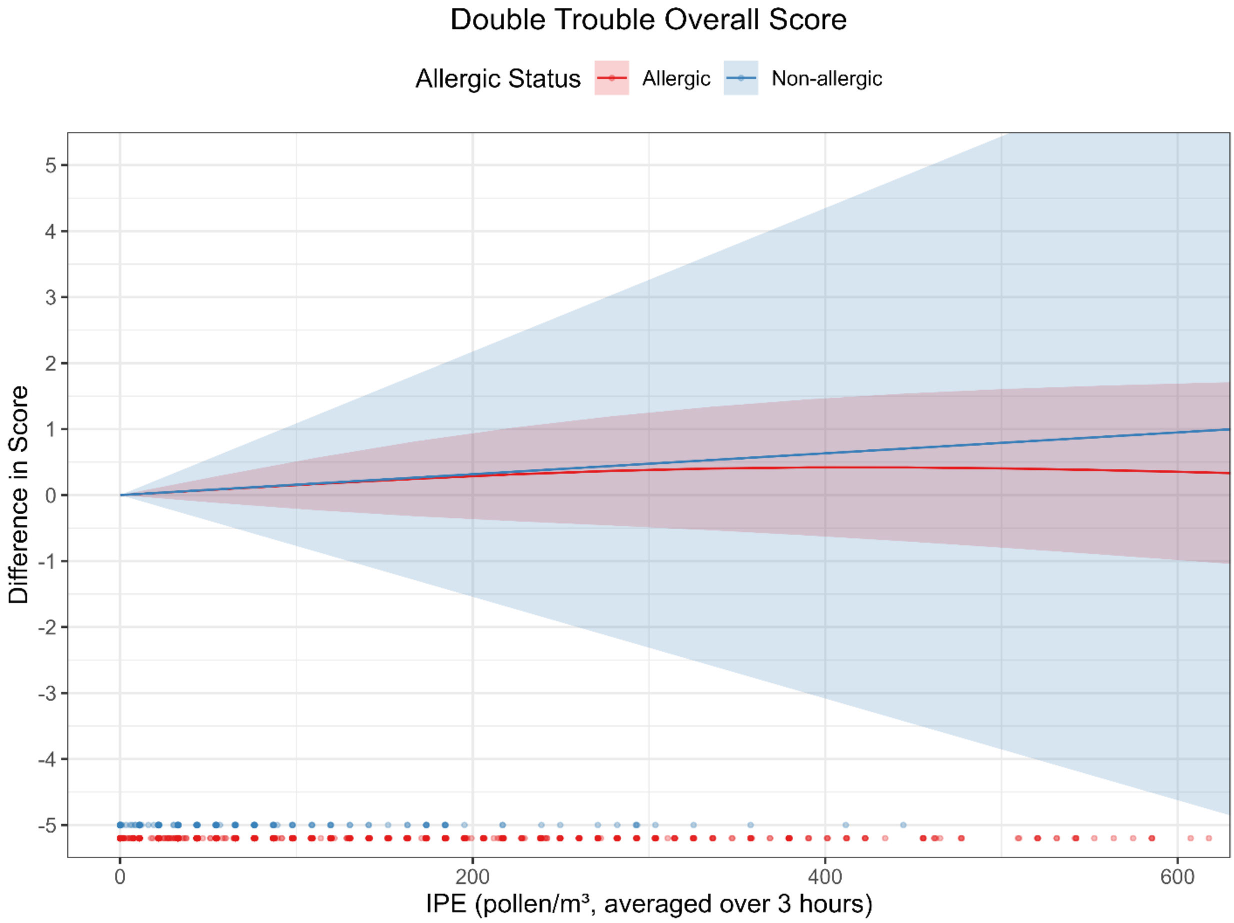

Figure S.4A: *Exposure-response plots stratified by allergic vs. non-allergic participants. The plot depicts the change in Double Trouble scores in relation to individually relevant pollen exposure (pollen/m³), averaged over the three hours preceding cognitive testing. Shaded regions indicate the 95% confidence intervals for each group. The bottom dotted lines show the distribution of observations for each group.*

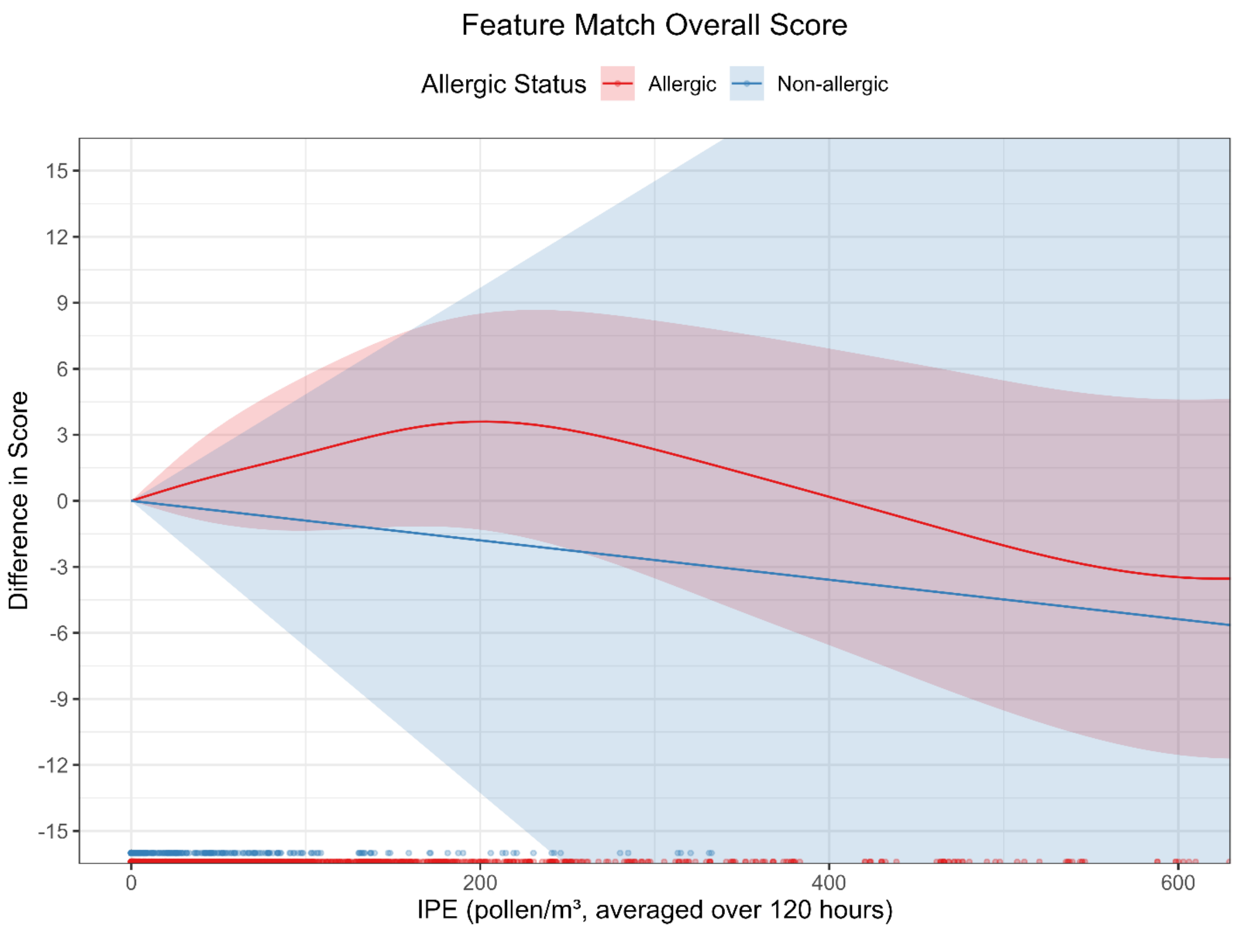

Figure S.4B: *Exposure-response plots stratified by allergic vs. non-allergic participants. The plot depicts the change in Feature Match scores in relation to individually relevant pollen exposure (pollen/m³), averaged over the 120 hours preceding cognitive testing. Shaded regions indicate the 95% confidence intervals for each group. The bottom dotted lines show the distribution of observations for each group.*

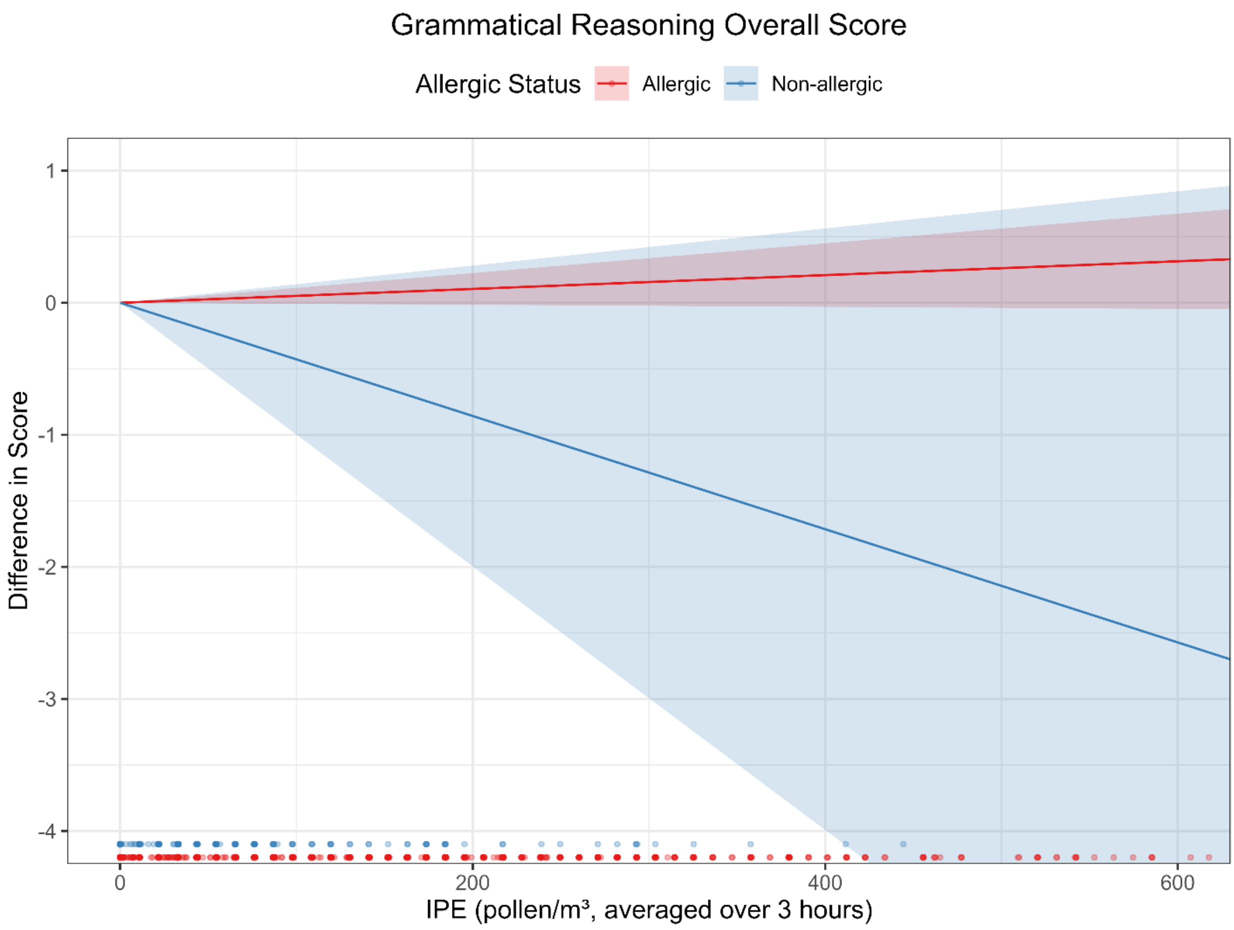

Figure S.4C: *Exposure-response plots stratified by allergic vs. non-allergic participants. The plot depicts the change in Grammatical Reasoning scores in relation to individually relevant pollen exposure (pollen/m³), averaged over the three hours preceding cognitive testing. Shaded regions indicate the 95% confidence intervals for each group. The bottom dotted lines show the distribution of observations for each group.*

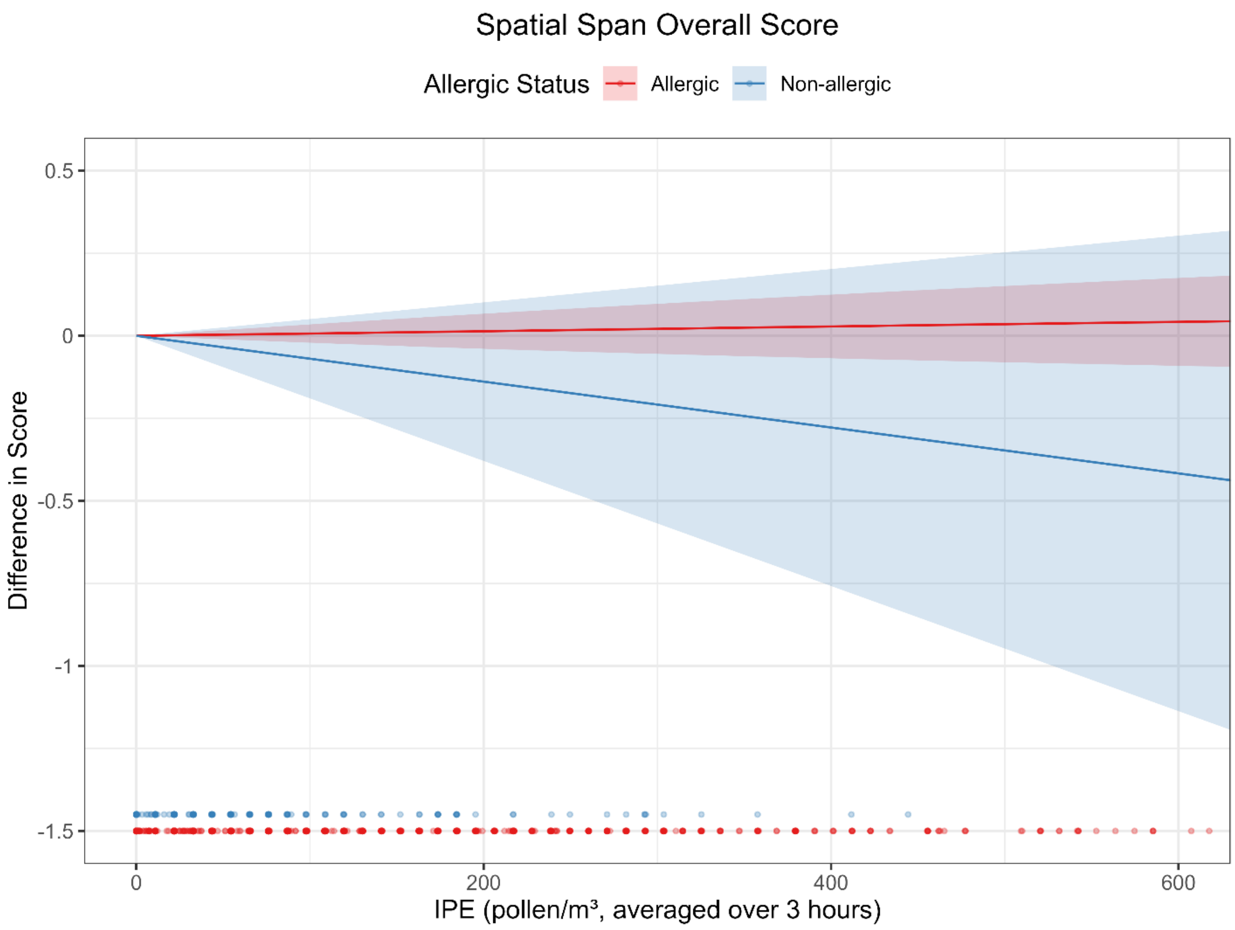

Figure S.4D: *Exposure-response plots stratified by allergic vs. non-allergic participants. The plot depicts the change in Spatial Span scores in relation to individually relevant pollen exposure (pollen/m³), averaged over the three hours preceding cognitive testing. Shaded regions indicate the 95% confidence intervals for each group. The bottom dotted lines show the distribution of observations for each group.*

S.5 Individual random intercept and population and individual learning effect on test score and reaction time

Task	Individual random intercept	Population average learning effect	Individual random slope	Absolute population average learning effect on day 10 [s]	Relative population average learning effect on day 10
**Double Trouble**	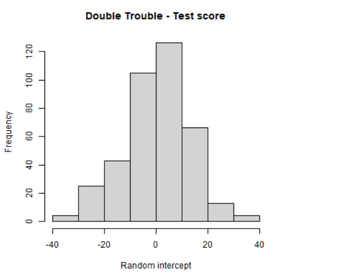	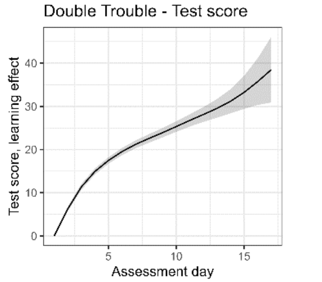	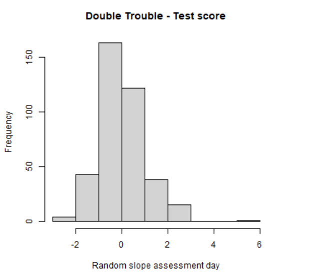	25.3 (95% CI: 24.1, 26.6)	55.1% (95% CI: 52.4%, 57.7%)
**Feature Match**	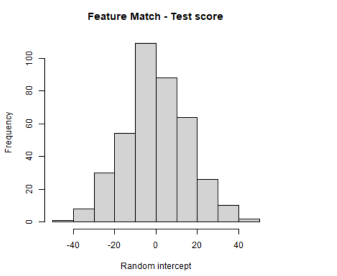	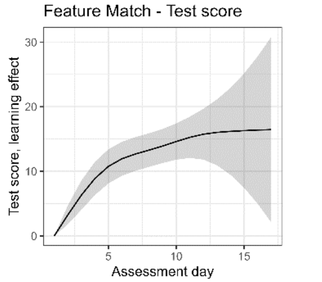	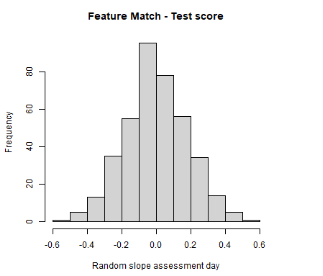	14.6 (95% CI: 11.8, 17.4)	10.5% (95% CI: 8.5%, 12.5%)
**Grammatical Reasoning**	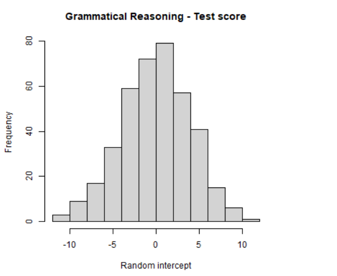	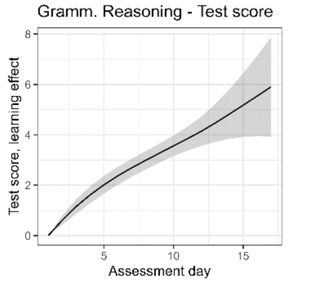	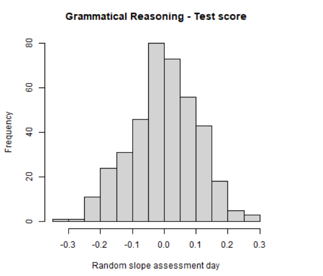	3.6 (95% CI: 3.2, 4.0)	19.2% (95% CI: 17.0%, 21.3%)
**Spatial Span**	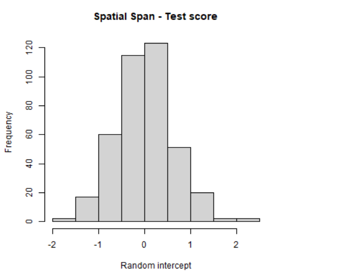	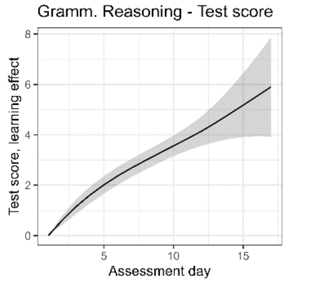	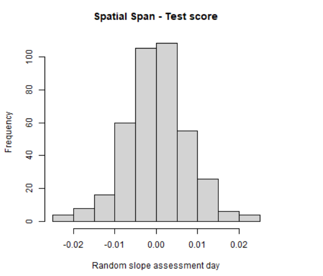	0.31 (95% CI: 0.24, 0.39)	5.3% (95% CI: 4.0%, 6.6%)

Figure S.5A: *Visualization of the individual random intercept in test score, and the learning effect on test score, as characterized by the population average and individual random slope. CI: Confidence Interval.*

Task	Individual random intercept	Population average learning effect	Individual random slope	Absolute population average learning effect on day 10 [s]	Relative population average learning effect on day 10
**Double Trouble**	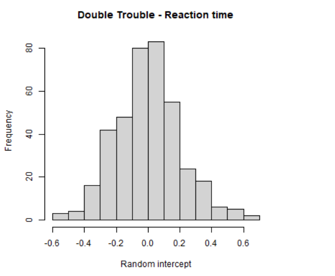	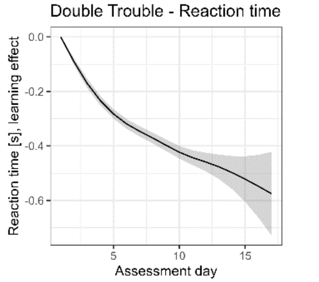	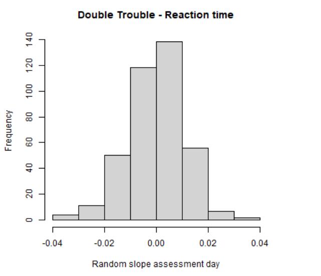	-0.42 (95% CI: -0.45, -0.40)	-22.5% (95% CI: -23.7%, -21.3%)
**Feature Match**	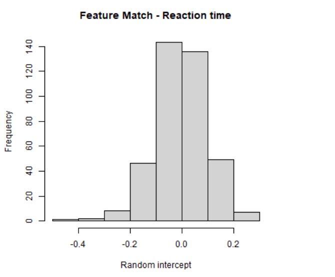	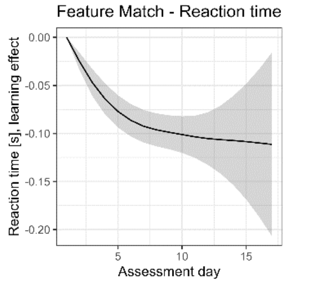	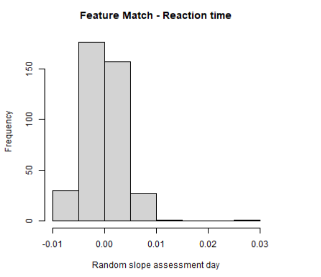	-0.10 (95% CI: -0.12, -0.08)	-3.4% (95% CI: -4.0%, -2.7%)
**Grammatical Reasoning**	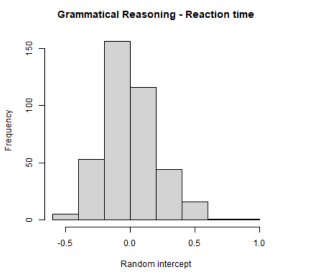	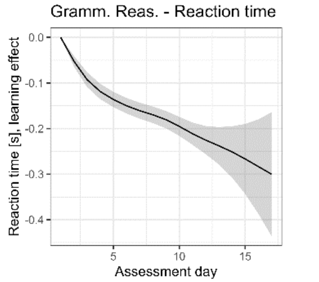	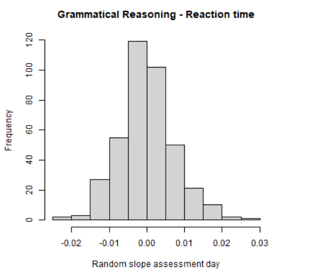	-0.20 (95% CI: -0.22, -0.17)	-5.5% (95% CI: -6.1%, -4.9%)
**Spatial Span**	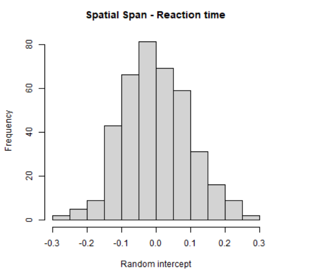	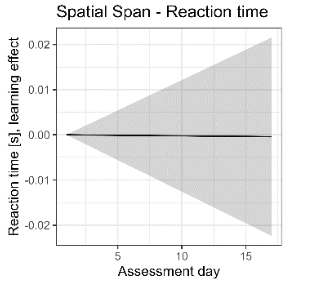	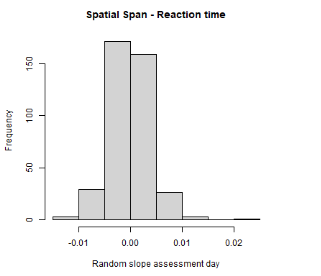	-0.00024 (95% confidence interval: -0.013, 0.012)	-0.0021% (95% CI: -0.11%, 0.11%)

Figure S.5B: *Visualization of the individual random intercept in reaction time, and the learning effect on reaction time, as characterized by the population average and individual random slope. CI: Confidence Interval.*

S.6 Difference in reaction time for each test using individually-relevant pollen exposure

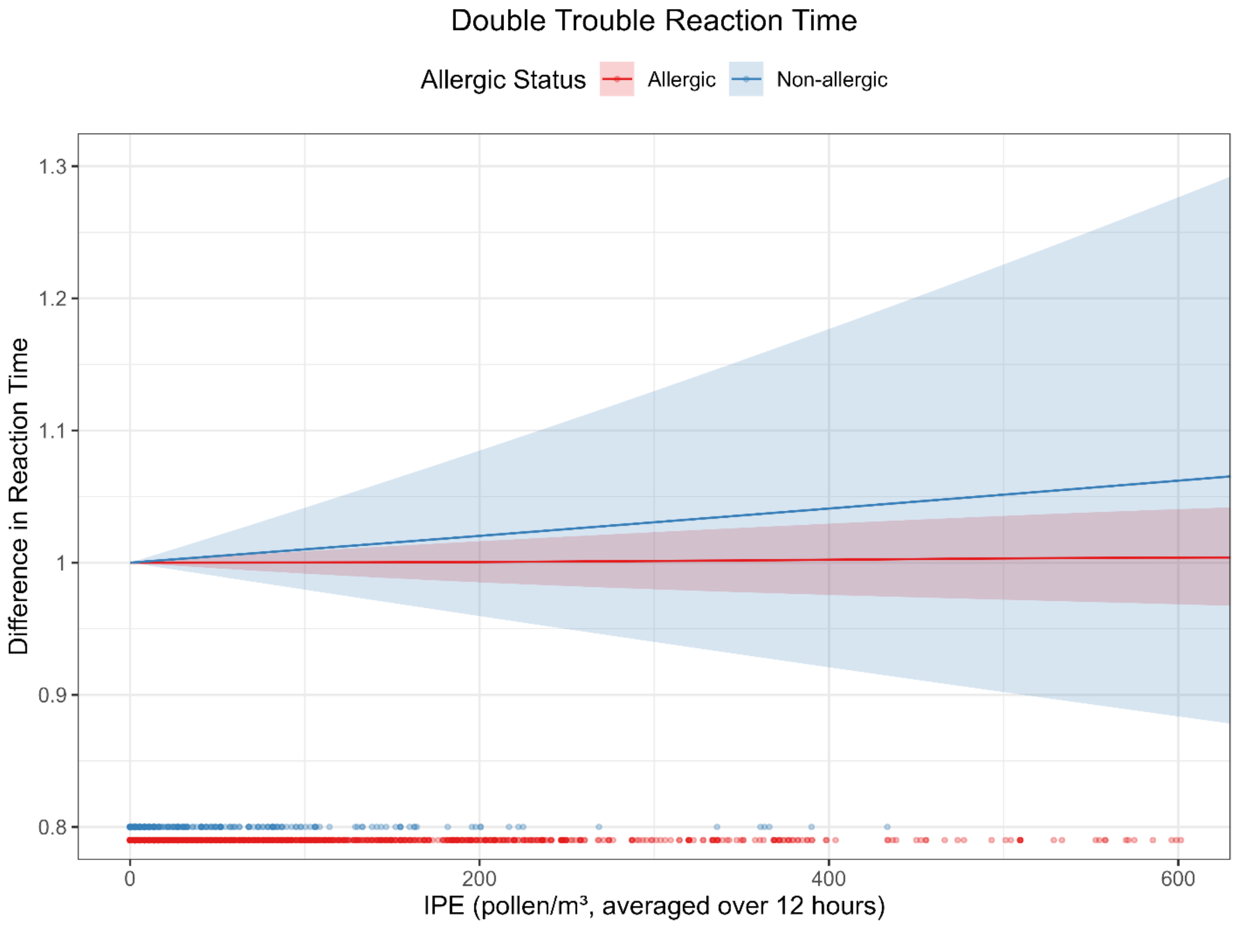

Figure S.6A: *Exposure-response plots stratified by allergic vs. non-allergic participants. The plot depicts the change in Double Trouble reaction time (on a multiplicative scale) in relation to individually relevant pollen exposure (pollen/m³), averaged over the 12 hours preceding cognitive testing. Shaded regions indicate the 95% confidence intervals for each group. The bottom dotted lines show the distribution of observations for each group.*

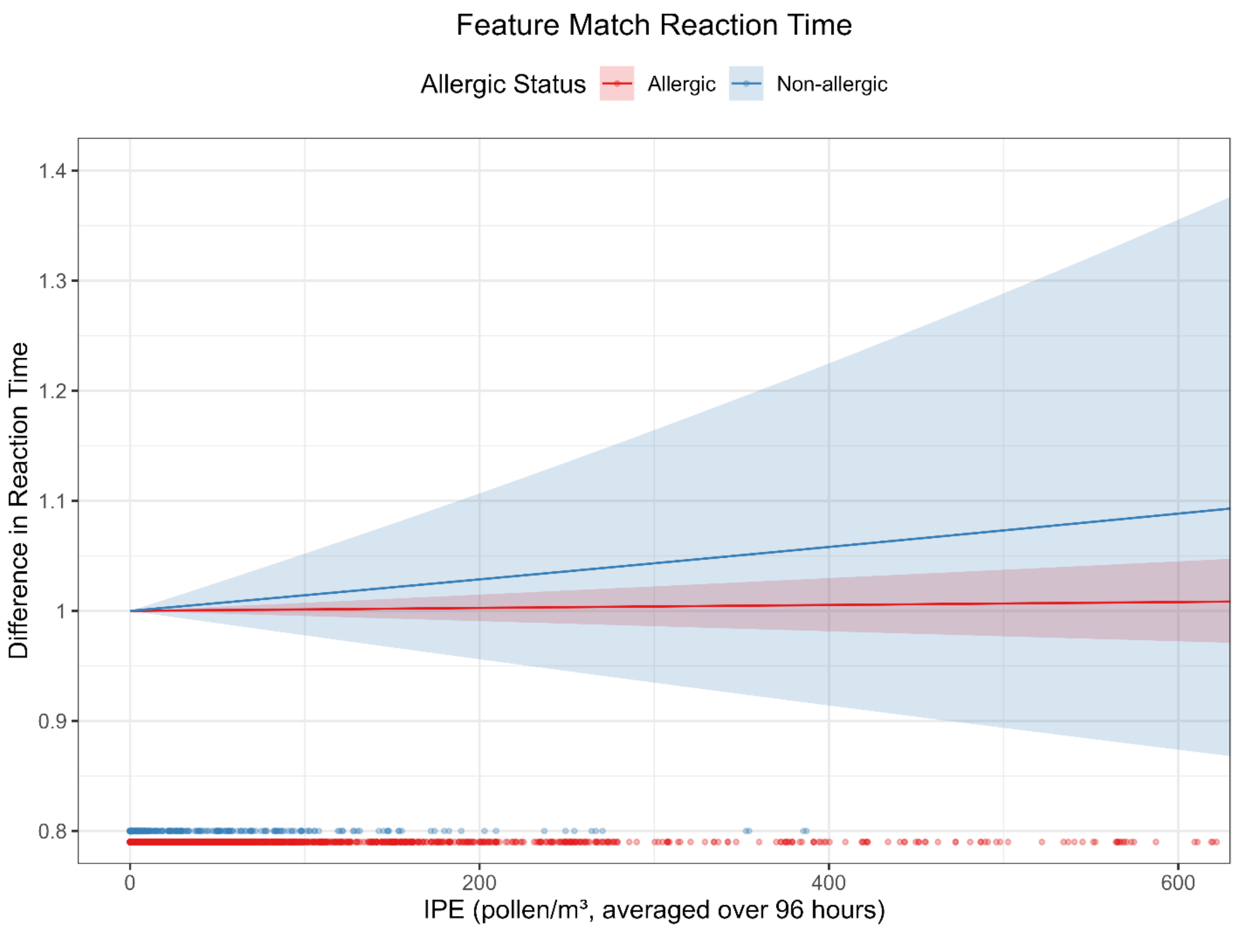

Figure S.6B: *Exposure-response plots stratified by allergic vs. non-allergic participants. The plot depicts the change in Feature Match reaction time (on a multiplicative scale) in relation to individually relevant pollen exposure (pollen/m³), averaged over the 96 hours preceding cognitive testing. Shaded regions indicate the 95% confidence intervals for each group. The bottom dotted lines show the distribution of observations for each group.*

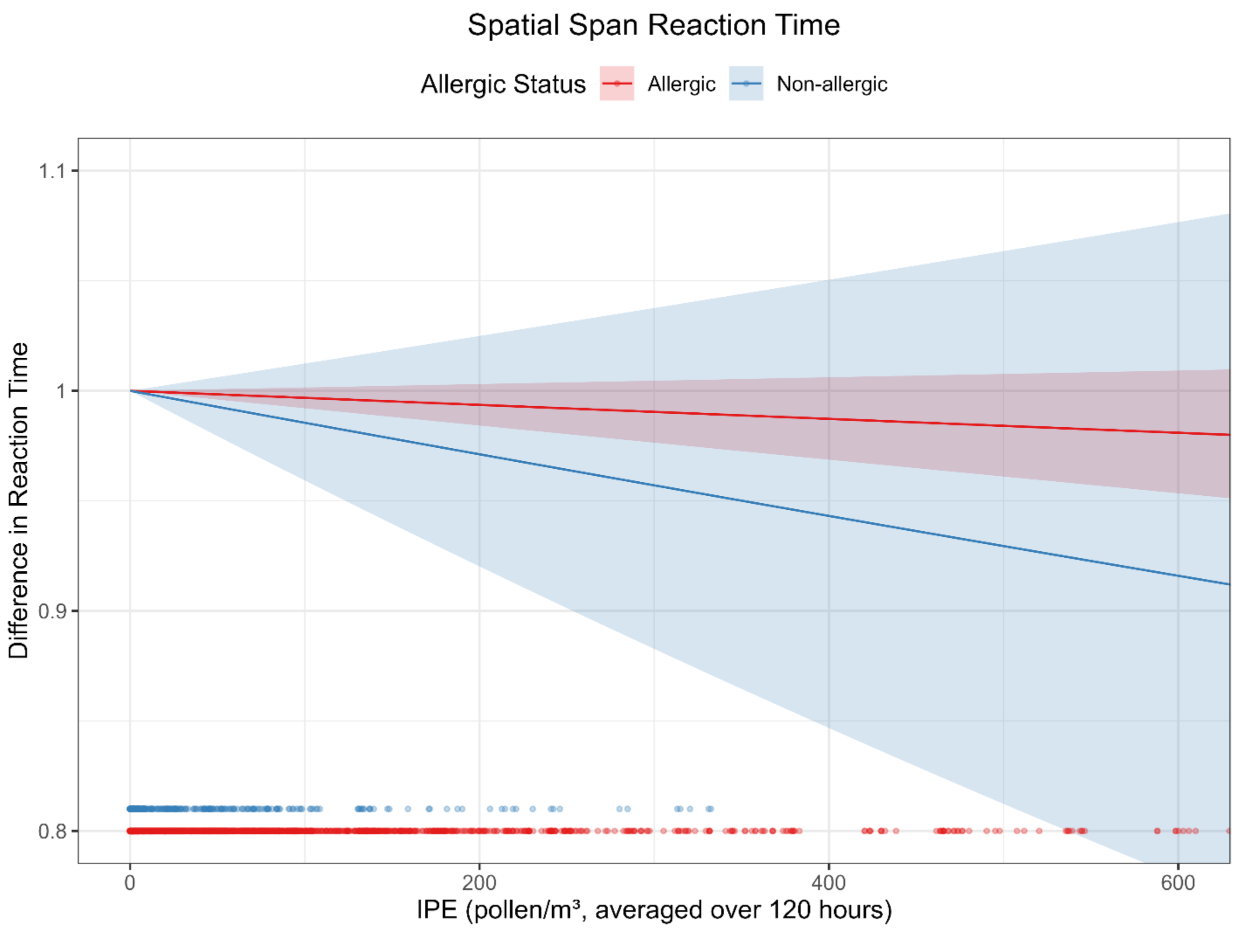

Figure S.6C: *Exposure-response plots stratified by allergic vs. non-allergic participants. The plot depicts the change in Spatial Span reaction time (on a multiplicative scale) in relation to individually relevant pollen exposure (pollen/m³), averaged over the 120 hours preceding cognitive testing. Shaded regions indicate the 95% confidence intervals for each group. The bottom dotted lines show the distribution of observations for each group.*

S.7 Difference in overall score in relation to symptom severity scores

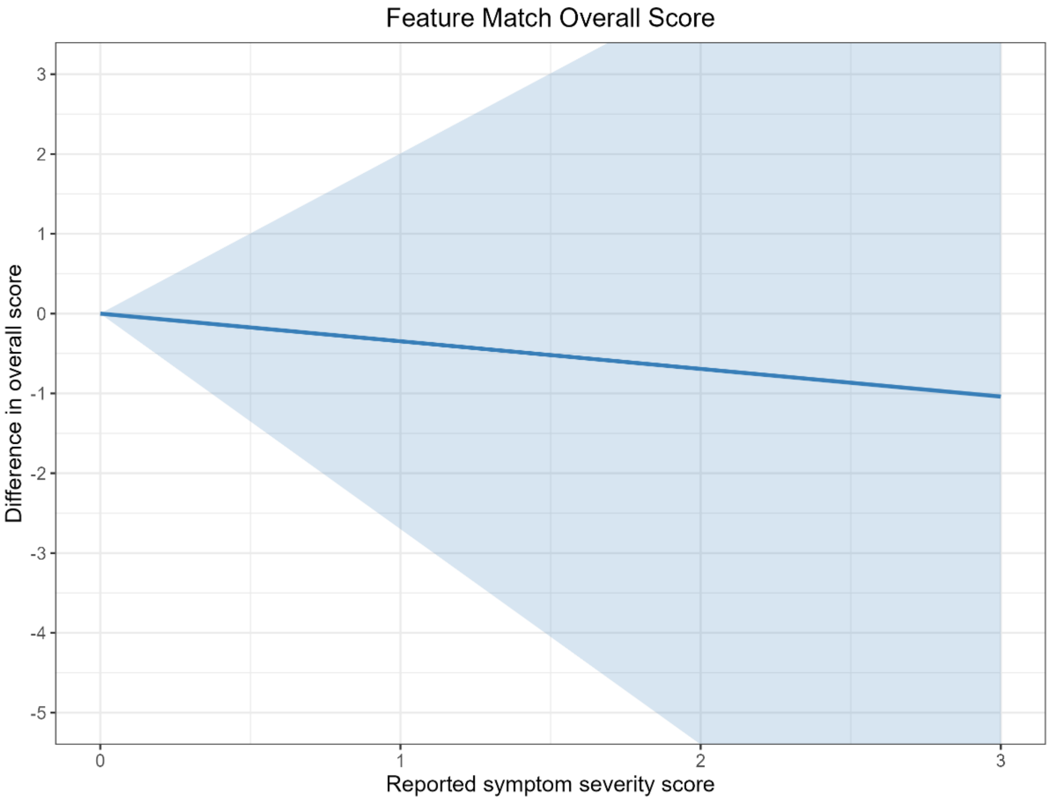

Figure S.7A: *Change in Feature Match scores in relation to self-reported nasal ocular allergic symptom scores. Shaded areas indicate the 95% confidence interval.*

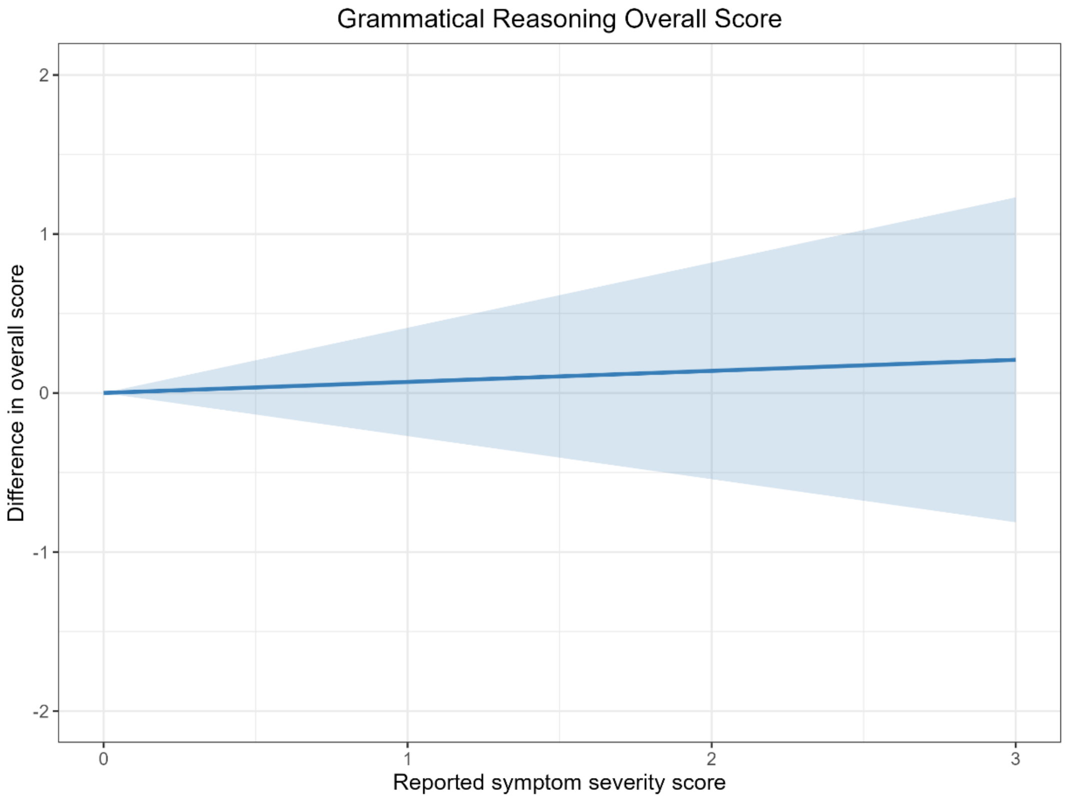

Figure S.7B: *Change in Grammatical Reasoning scores in relation to self-reported nasal ocular allergic symptom scores. Shaded areas indicate the 95% confidence interval.*

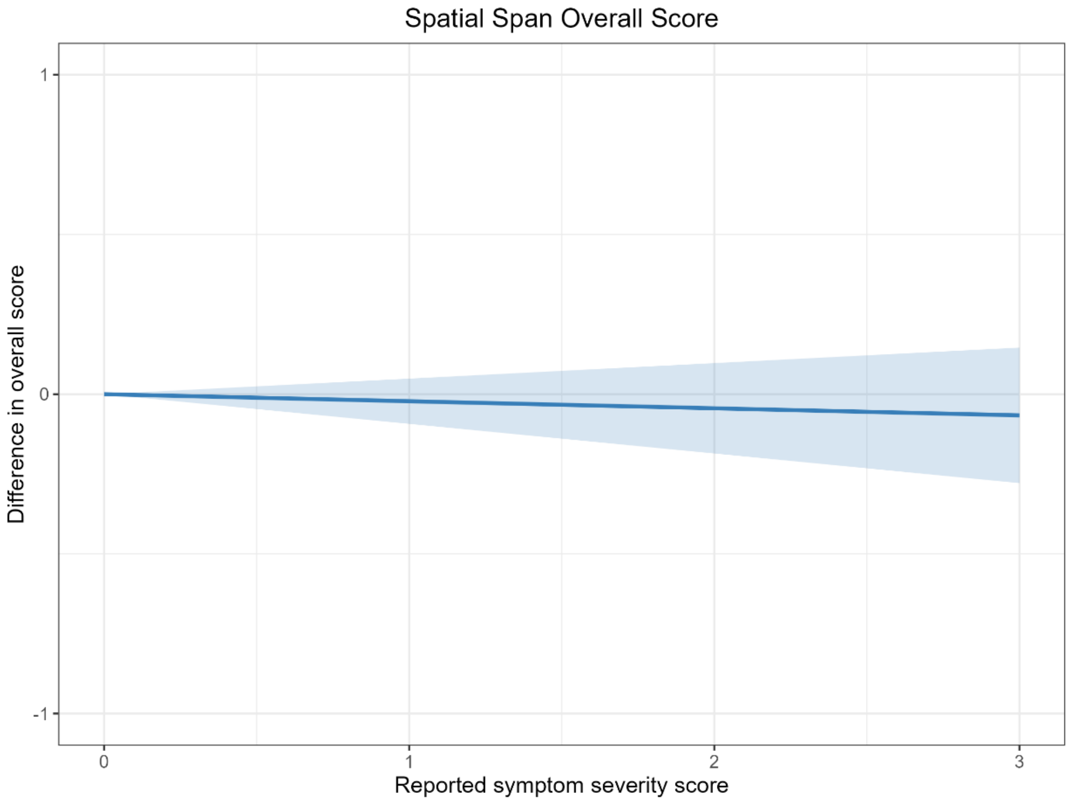

Figure S.7C: *Change in Spatial Span scores in relation to self-reported nasal ocular allergic symptom scores. Shaded areas indicate the 95% confidence interval.*

S.8 Difference in reaction time in relation to symptom severity scores

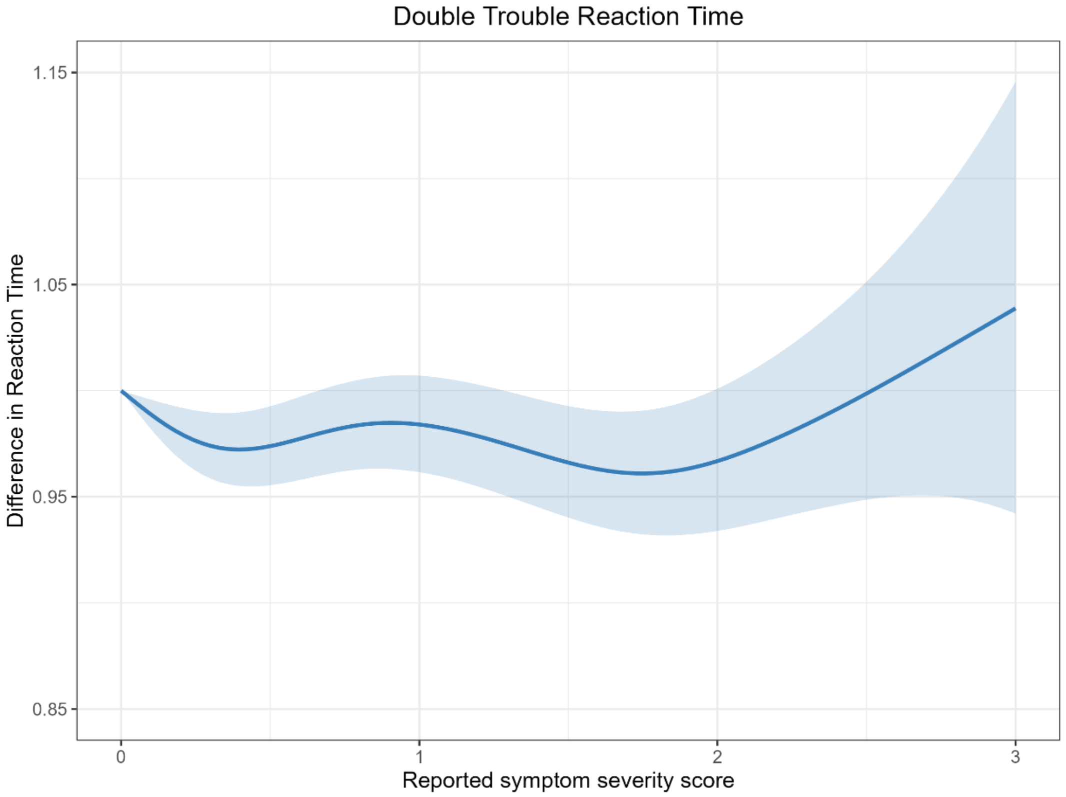

Figure S.8A: *Change in Double Trouble reaction time (milliseconds, multiplicative scale) in relation to self-reported nasal ocular allergic symptom scores. Shaded areas indicate the 95% confidence interval.*

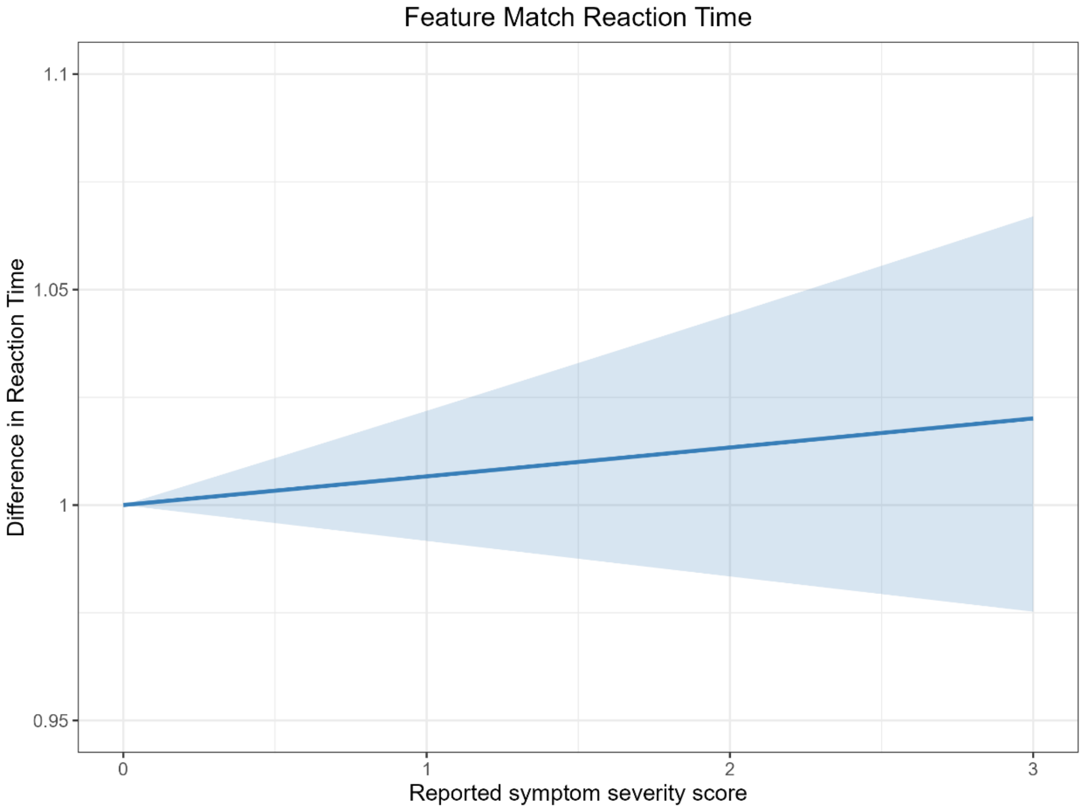

Figure S.8B: *Change in Feature Match reaction time (milliseconds, multiplicative scale) in relation to self-reported nasal ocular allergic symptom scores. Shaded areas indicate the 95% confidence interval.*

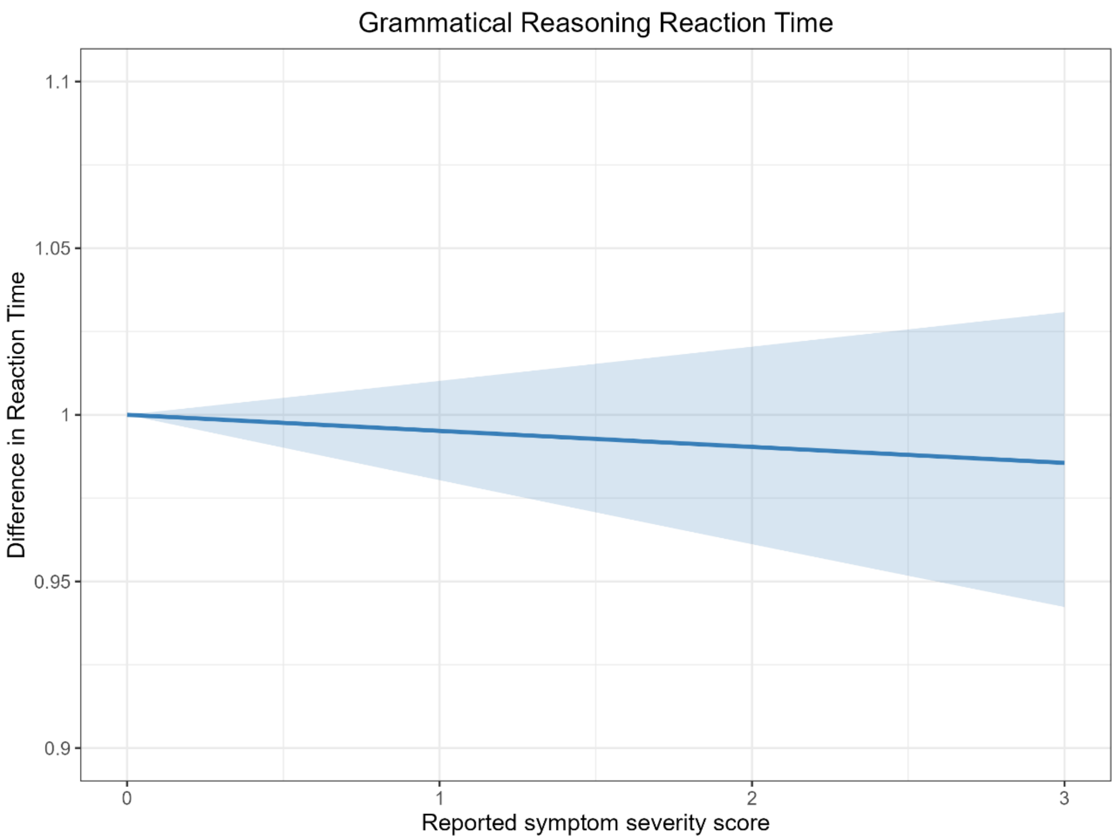

Figure S.8C: *Change in Grammatical Reasoning reaction time (milliseconds, multiplicative scale) in relation to self-reported nasal ocular allergic symptom scores. Shaded areas indicate the 95% confidence interval.*

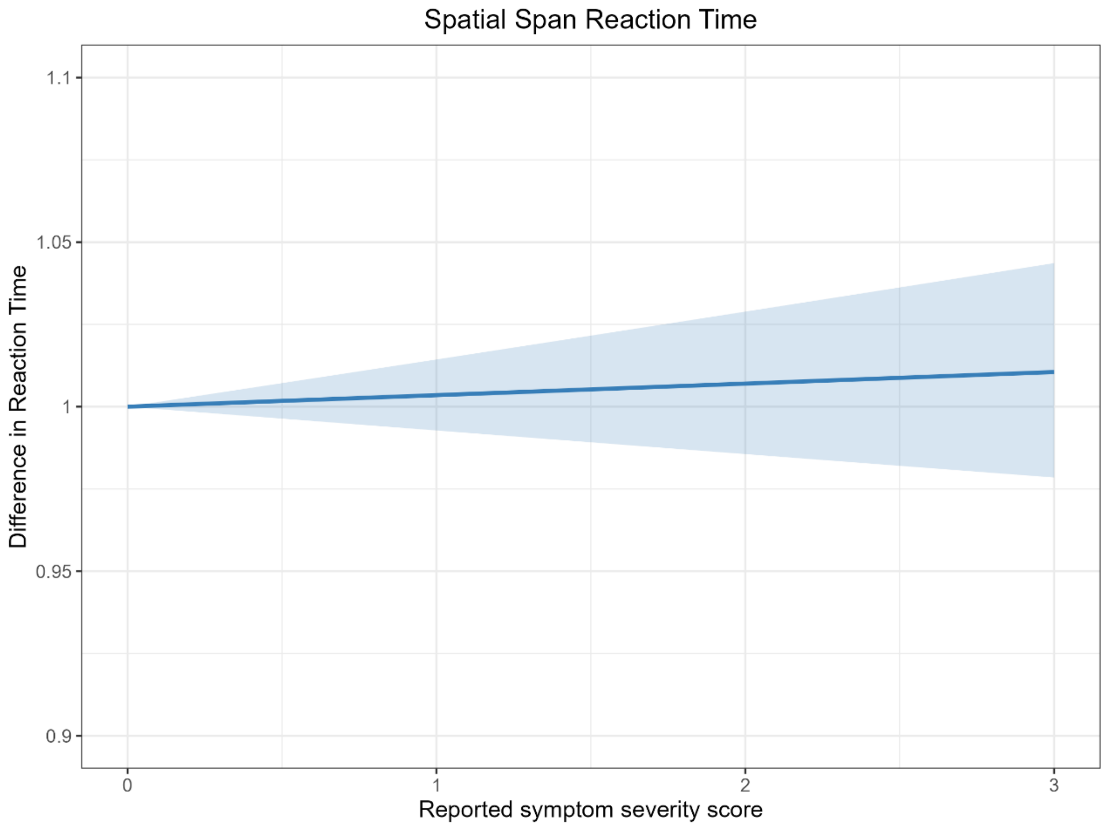

Figure S.8D: *Change in Spatial Span reaction time (milliseconds, multiplicative scale) in relation to self-reported nasal ocular allergic symptom scores. Shaded areas indicate the 95% confidence interval.*

References

1. Stroop JR. Studies of interference in serial verbal reactions. *Journal of experimental psychology* 1935;**18**(6):643.
2. Hampshire A, Thompson R, Duncan J, Owen AM. Selective tuning of the right inferior frontal gyrus during target detection. *Cognitive, Affective, & Behavioral Neuroscience* 2009;**9**(1):103-112.
3. Baddeley AD. A 3 min reasoning test based on grammatical transformation. *Psychonomic science* 1968;**10**(10):341-342.
4. Kessels RP, Van Zandvoort MJ, Postma A, Kappelle LJ, De Haan EH. The Corsi block-tapping task: standardization and normative data. *Applied neuropsychology* 2000;**7**(4):252-258.
5. Bürgler A, Luyten A, Glick S, et al. Association between short-term pollen exposure and blood pressure in adults: a repeated-measures study. *Environmental Research* 2024:119224.
